# Risk of Serious Adverse Events and Death With Low‐Dose Methotrexate Versus Hydroxychloroquine in Adults Receiving Dialysis

**DOI:** 10.1111/sdi.70035

**Published:** 2026-05-12

**Authors:** Flory T. Muanda, Peter G. Blake, Matthew A. Weir, Fatemeh Ahmadi, Mohammad Ali Omrani, Sheikh S. Abdullah, Eric McArthur, Jessica M. Sontrop, Brad L. Urquhart, Amit X. Garg

**Affiliations:** ^1^ ICES Ontario Canada; ^2^ Department of Physiology and Pharmacology, Schulich School of Medicine and Dentistry Western University London Ontario Canada; ^3^ Department of Epidemiology and Biostatistics, Schulich School of Medicine and Dentistry Western University London Ontario Canada; ^4^ Division of Nephrology, Department of Medicine, Schulich School of Medicine and Dentistry Western University London Ontario Canada; ^5^ London Health Sciences Centre Research Institute, London Health Sciences Centre, Schulich School of Medicine and Dentistry Western University London Ontario Canada

**Keywords:** dialysis, dosage, hydroxychloroquine, low‐dose methotrexate, toxicity

## Abstract

**Background:**

Several case reports have linked low‐dose methotrexate to serious adverse events, including death, in dialysis patients. We compared the risk of serious adverse events in dialysis patients initiating low‐dose methotrexate versus hydroxychloroquine.

**Methods:**

Using linked healthcare databases in Ontario, Canada (1997–2020), we identified 55 new users of low‐dose methotrexate and 407 new users of hydroxychloroquine.

The primary outcome was the 90‐day risk of death or hospitalization with myelosuppression, sepsis, pneumotoxicity, or hepatotoxicity. Adjusting for age, sex, and a proxy for polypharmacy, a modified Poisson regression was used to estimate adjusted risk ratios (aRR), and a binomial regression was used to estimate adjusted risk differences (aRD).

**Results:**

The median prescribed dose was 10 mg/week (IQR, 10–17.5) for methotrexate and 300 mg/day (IQR, 200–400) for hydroxychloroquine. The primary outcome occurred in 16/55 low‐dose methotrexate users (29.1%) and in 29/407 hydroxychloroquine users (7.1%); aRR: 3.14 (95% CI, 1.75 to 5.63); aRD: 19.5% (95% CI, 7.5% to 31.4%). Findings were consistent across sensitivity analyses.

**Conclusion:**

Low‐dose methotrexate should be avoided in dialysis patients whenever possible, and alternative DMARDs should be considered.

## 1 Introduction

Low‐dose methotrexate is a disease‐modifying antirheumatic drug (DMARD) primarily used for the treatment of rheumatoid arthritis and psoriasis [1, 2, 3]. It is also prescribed as an immunomodulator for adults with Crohn's disease [1, 2, 3]. The usual dose of methotrexate for these conditions is 5–25 mg per week [1, 2, 3] and occasionally, the maximum weekly dose is increased to 35 mg [1, 2, 3]. Low‐dose methotrexate is also prescribed off‐label for dermatomyositis, eczema, systemic lupus erythematosus, and systemic sclerosis [1, 2, 3]. In the United States, 4.5 million low‐dose methotrexate prescriptions were dispensed in 2022 [4].

Methotrexate is extensively cleared by the kidneys, so a dose reduction is recommended in patients with reduced kidney function. In a previous study, we found that older adults with chronic kidney disease (CKD) who started low‐dose methotrexate were twice as likely to be hospitalized with myelosuppression, sepsis, or hepatotoxicity compared with matched patients who started hydroxychloroquine (a different DMARD with indications similar to low‐dose methotrexate) [5]. Among those with the lowest levels of kidney function (estimated glomerular filtration rate [eGFR] less than 45 mL/min/1.73 m^2^), one case of a serious adverse event occurred for every 28 adults prescribed low‐dose methotrexate, further reinforcing the risks associated with low‐dose methotrexate use in older adults with low kidney function.

Our literature search identified 22 case reports describing adverse events associated with low‐dose methotrexate in adults receiving dialysis (search strategy in Table S1; summary of studies in Table S2); the most serious adverse event reported was pancytopenia. Further, in several high‐profile coroner's cases, low‐dose methotrexate was linked to severe adverse effects in adults with low kidney function [6, 7]. To date, no population‐based studies have evaluated the safety of low‐dose methotrexate in adults receiving maintenance dialysis.

To address this, we conducted a population‐based study to compare the 90‐day risk of death or a hospital visit with myelosuppression, sepsis, pneumotoxicity, or hepatotoxicity in adults receiving maintenance dialysis who started low‐dose methotrexate versus hydroxychloroquine.

## 2 Methods

### 2.1 Study Design and Setting

This study was conducted using linked administrative healthcare databases in the province of Ontario, Canada (1997–2020). These databases contain encrypted data on healthcare visits and hospitalizations for all residents (~15 million). All residents have universal access to hospital care and physician services through a government‐funded single‐payer system [8]. In Ontario, approximately 12,000 adults receive outpatient maintenance dialysis [9], and about 90% of these patients have universal prescription drug coverage through the Ontario Drug Benefit (ODB) plan; prescription drugs dispensed to outpatients covered by ODB are recorded in the ODB database. The use of data in this study was authorized under Section 45 of Ontario's Personal Health Information Protection Act, which does not require review by a Research Ethics Board or individual informed consent. Study reporting follows recommended guidelines for pharmaco‐epidemiological studies that use routinely collected health data (Supplementary Table S3) [10].

### 2.2 Data Sources and Variables

Data for this study were obtained from seven healthcare databases housed at ICES (ices.on.ca) [11]. The following datasets were linked using unique encoded identifiers and analyzed at ICES: The Canadian Institute for Health Information Discharge Abstract Database, the ICES‐derived Physician Database, the National Ambulatory Care Reporting System database, the (ODB) Database, the Ontario Health Insurance Plan database, the Ontario Mental Health Reporting System Database, and the Registered Persons Database. Data on hospital admissions and diagnoses were coded by trained personnel using the *International Classification of Diseases 10th revision* (ICD‐10) system; personnel only consider physician‐recorded diagnoses in a patient's medical chart when assigning codes, and do not review or interpret symptoms or test results. We have previously utilized these databases to investigate prescription drug safety [5, 12, 13, 14].

The databases were complete for all variables in this study except for data on prescriber specialty (19% missing, handled using a missing indicator approach and coded as a separate “unknown” category), neighborhood income quintile (0.4% missing, recorded as the middle quintile), and urban or rural location of residence (< 0.01% missing, re‐coded as urban location of residence). The only reason for loss to follow‐up was emigration from the province, which is < 0.5% per year on average [15]. The codes used to ascertain comorbidities and outcomes are detailed in Table S4. We assessed comorbidities in the 5‐year period before cohort entry and healthcare use in the 1‐year period before cohort entry. We used a 120‐day look‐back period to ascertain the use of other prescription drugs because the Ontario Drug Benefit program allows a maximum prescription duration of 100 days.

### 2.3 Patients

We selected adults aged 18 years and older who were receiving maintenance dialysis (including hemodialysis and peritoneal dialysis, as defined in Supplementary Table S5) and who were newly dispensed oral methotrexate or hydroxychloroquine from an outpatient pharmacy between January 1, 1997, and December 31, 2020. The prescription fill date was the patient's cohort entry date; adults could only enter the cohort once. To ensure eligibility for provincial universal prescription drug coverage, we included only adults who had received at least one outpatient drug prescription in the 120 days before cohort entry. We excluded adults aged < 18 years, as the study aimed to assess the safety of low‐dose methotrexate in adults receiving maintenance dialysis, and the management and outcomes of dialysis treatment may differ importantly between children and adults. To ensure that adults were new users and receiving monotherapy at treatment initiation, we excluded individuals with any evidence of methotrexate or hydroxychloroquine use in the 180 days before the cohort entry date, those with a prescription for another DMARD (conventional or biologic) on the cohort entry date, and those with more than one methotrexate or hydroxychloroquine prescription on the cohort entry date, thereby ensuring monotherapy initiation of low‐dose methotrexate or hydroxychloroquine. We also excluded adults who were discharged from the hospital or emergency department within 2 days before cohort entry (in Ontario, adults who start a DMARD prescription during a hospital admission would have their outpatient prescription dispensed on the same day or the day after hospital discharge). To ensure generalizability to usual prescribing, we excluded adults who started methotrexate or hydroxychloroquine at non‐standard doses (i.e., methotrexate < 5 mg/week or > 35 mg/week; hydroxychloroquine < 200 mg/day or > 400 mg/day). A diagram summarizing the cohort creation steps is provided in Figure S1.

### 2.4 Exposure

The exposure was low‐dose oral methotrexate (5–35 mg/week). Oral hydroxychloroquine (200–400 mg/day) was selected as an active comparator to minimize indication bias, consistent with our prior study [5]. As a DMARD, hydroxychloroquine shares several indications with methotrexate, including rheumatoid arthritis, systemic lupus erythematosus, and dermatomyositis [14]. Using an active‐comparator design helps ensure that patients have similar treatment indications and disease severity, thereby reducing confounding that may occur when comparing treated patients with those not receiving DMARD therapy [16].

### 2.5 Outcomes

All primary and secondary outcomes were pre‐specified. The primary outcome was the 90‐day risk of death or a hospital visit (i.e., an emergency department visit or a hospital admission) with myelosuppression (defined as a diagnosis of aplastic anemia, neutropenia, thrombocytopenia, or pancytopenia), sepsis, pneumotoxicity, or hepatotoxicity. These adverse events, which often occurred within the first few weeks of methotrexate initiation, have been reported in several randomized clinical trials of low‐dose methotrexate [17], in our previous study involving patients with CKD [5], and in case reports of patients receiving dialysis (Table S2). To evaluate this risk, and consistent with our prior CKD study, we chose 90 days to focus on acute events and to avoid crossovers or drug discontinuation that could occur with more extended periods of follow‐up. The secondary outcomes were the components of the primary composite outcome and all‐cause hospitalization. Diagnostic codes for all outcome variables and information on their validation and interpretation are provided in Table S6.

### 2.6 Statistical Analysis

Analyses were conducted using SAS version 9.4 (SAS Institute, Cary, NC). We initially planned to use propensity score matching to balance the comparison groups on 95 indicators of baseline health, including all known indications for methotrexate use (both on‐label and off‐label indications as listed in Table 1 and/or Table S7). However, because of the small sample of methotrexate users, the regression analyses for the primary and secondary outcomes were adjusted for only three key risk factors—age (years), sex (male/female), and the number of unique drugs prescribed in the 120 days before cohort entry (total count; a proxy for polypharmacy)—to reduce the risk of overfitting. These covariates were chosen because they are well‐established, clinically relevant predictors of the study outcomes, including mortality, myelosuppression, sepsis, pneumotoxicity, and hepatotoxicity [18, 19, 20]. This approach aligns with the recommendation to limit the number of covariates relative to the number of outcome events (e.g., 10 events per variable [EPV]) [21]. In our study, this corresponded to 15 EPV across 45 primary outcome events.

**TABLE 1:** Baseline characteristics^a^ of adults receiving maintenance dialysis newly prescribed low‐dose methotrexate vs. hydroxychloroquine in Ontario, Canada (1997–2020).

	Low‐dose methotrexate (*n* = 55)	Hydroxychloroquine (*n* = 407)	Standardized difference^b^
Demographics			
Women, no. (%)	28 (50.9)	291 (71.5)	43%
Men, no. (%)	27 (49.1)	116 (28.5)	43%
Age, mean (SD), y	63.2 (15.0)	54.9 (18.2)	49%
Year of cohort entry, no. (%)			
1997–2004	8 (14.5)	63 (15.5)	3%
2005–2012	19 (34.5)	129 (31.7)	6%
2013–2020	28 (50.9)	215 (52.8)	4%
Income quintile, no. (%)^c^			
1 (lowest)	9 (16.4)	110 (27.0)	26%
2	10 (18.2)	105 (25.8)	18%
3 (middle)	12 (21.8)	82 (20.1)	4%
4	15 (27.3)	61 (15.0)	3%
5 (highest)	9 (16.4)	49 (12.0)	13%
Dialysis modality, *n* (%)			
Hemodialysis	51 (91.7)	366 (89.9)	10%
Prescriber specialty, no. (%)			
Rheumatologist	27 (49.1)	182 (44.7)	9%
Other	16 (29.1)	149 (36.6)	16%
Missing	12 (21.8)	76 (18.7)	8%
Comorbidities, no. (%)^d^			
Rheumatoid arthritis	27 (49.1)	175 (43.0)	12%
Atopic dermatitis or eczema	18 (32.7)	140 (34.4)	4%
Psoriasis	13 (23.6)	11 (2.7)	65%
Systemic lupus erythematosus	13 (23.6)	223 (54.8)	67%
Dermatomyositis	11 (20.0)	202 (49.6)	65%
Modified Charlson comorbidity index, mean (SD)^e^	5.1 (2.4)	5.1 (2.1)	2%
Healthcare visits/tests^f^			
Primary care visits, mean (SD)	12.9 (15.2)	11.1 (13.0)	13%
Emergency department visits, mean (SD)	2.3 (2.8)	2.6 (3.2)	11%
Medication use, no. (%)^g^			
Statins	20 (36.4)	78 (18.4)	41%
Beta‐blockers	20 (36.4)	61 (15.0)	51%
Number of unique medications, mean (SD)^h^	7.2 (8.1)	4.0 (6.0)	46%

Abbreviations: IQR, interquartile range; SD, standard deviation.

^a^ Unless otherwise specified in the footnotes, baseline characteristics were assessed on the date the patient filled the low‐dose methotrexate prescription or the hydroxychloroquine prescription—the cohort entry date.

^b^ The difference between the groups divided by the pooled SD; a value greater than 10% is interpreted as a meaningful difference.

^c^ Income was categorized into fifths of average neighborhood income at cohort entry.

^d^ Baseline comorbidities were assessed in the 5‐year period before cohort entry.

^e^ Presence of kidney disease is a variable in the Charlson comorbidity index, which automatically results in all individuals receiving a minimum score of 2.

^f^ Total number of healthcare visits/tests in the 12‐month period before cohort entry.

^g^ Medication use was examined in the 120‐day period before cohort entry (the Ontario Drug Benefit program dispenses a maximum 100‐day supply).

^h^ Total number of unique drugs prescribed in the 120‐day period before cohort entry.

Between‐group differences in baseline characteristics were examined using standardized mean differences (SMDs); SMDs greater than 10% are generally considered clinically significant. Unadjusted and adjusted risk ratios (RR and aRR) and 95% confidence intervals (CIs) were obtained using modified Poisson regression [22], while unadjusted and adjusted risk differences (RD and aRD) and 95% CIs were obtained using a binomial regression model with an identity link function. We calculated the unadjusted and adjusted number needed to harm (NNH and aNNH) as the reciprocal of the risk difference (1/RD and 1/aRD). The NNH is a measure that indicates how many patients need to receive low‐dose methotrexate for one patient to experience the harmful outcome, who would otherwise not have been harmed if they had received hydroxychloroquine (a lower number indicating greater harm). All analyses followed an intention‐to‐treat approach, classifying patients according to their initial DMARD at cohort entry and following them for 90 days regardless of subsequent treatment changes, including discontinuation or switching. We interpreted two‐tailed *p* values < 0.05 as statistically significant. To comply with ICES privacy regulations to minimize the risk of identification, values of cells with ≤ 5 adults were reported as < 6. We conducted four post hoc analyses to assess the robustness of our findings and potential sources of bias. First, to address concerns about overfitting and limited statistical power due to the small number of outcome events, we used a random forest algorithm to identify the most predictive covariates [23]. The most important variables identified were the exposure (low‐dose methotrexate vs. hydroxychloroquine), the total number of unique drugs prescribed before cohort entry, the total number of unique Drug Identification Numbers (DINs) before cohort entry, and the prescriber specialty. Because of substantial collinearity between the total number of unique drugs and the total number of DINs (variance inflation factor [VIF] > 75), only the total number of unique drugs was retained, and the final model included these three variables (~11 EPV). Second, we calculated E‐values to evaluate the potential impact of unmeasured confounding [24]. Third, we conducted a negative‐control outcome analysis using thyroid testing [25], which shares sources of bias with the primary outcome but is unlikely to be causally related to either exposure, to assess residual confounding [26, 27, 28, 29]. Finally, we performed an additional analysis, adjusting for 55 baseline variables with standardized differences > 10% (~0.8 EPV), to provide an additional assessment of the stability of effect estimates despite potential overfitting [21].

## 3 Results

**FIGURE 1:**
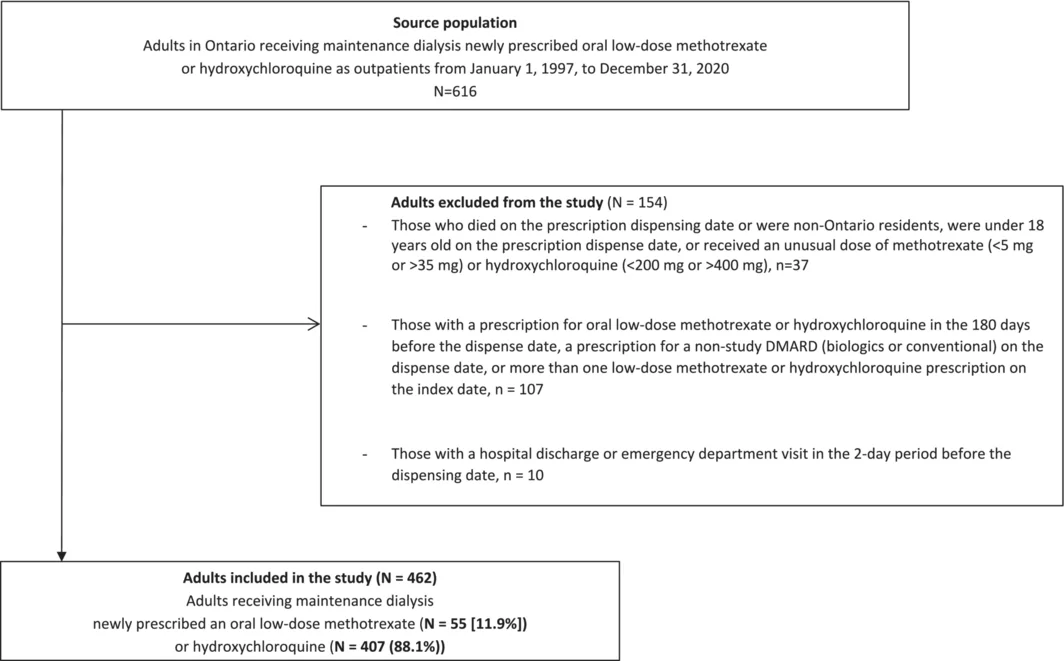
Flow diagram of cohort build. Abbreviation: DMARD, disease‐modifying antirheumatic drug.

The flow diagram for the cohort build is shown in Figure 1. After applying exclusions, the cohort included 462 adults receiving maintenance dialysis who were newly dispensed low‐dose methotrexate (*n* = 55) or hydroxychloroquine (*n* = 407) at an outpatient pharmacy. The median prescribed dose of methotrexate was 10 mg/week (IQR, 10–17.5), and it was 300 mg/day (IQR, 200–400) for hydroxychloroquine. The median duration of continuous dispensing was 28 days (IQR, 28–56) and 30 days (IQR, 30–90), respectively. At baseline, low‐dose methotrexate users differed from hydroxychloroquine users on many characteristics (Table 1 and Table S7). In particular, low‐dose methotrexate users were older (mean age 63 vs. 55 years, respectively), 50% were male compared with 29% of hydroxychloroquine users, a higher proportion had psoriasis (24% vs. 3%), and a lower proportion had systemic lupus erythematosus (24% vs. 55%) and dermatomyositis (20% vs. 50%); a higher proportion were receiving hemodialysis (93% vs. 90%); a higher proportion used statins (36% vs. 18%) and beta‐blockers (36% vs. 15%), and the mean number of unique drugs prescribed before cohort entry was 7 vs. 4.

The proportion of prescriptions issued by rheumatologists was similar between groups (49% vs. 45%). The number of adults receiving maintenance dialysis who were newly prescribed low‐dose methotrexate increased over time: 8 in 1997–2004, 19 in 2005–2012, and 28 in 2013–2020. While this reflects an increase in the absolute number of new prescriptions, the proportion of new users of low‐dose methotrexate entering the cohort within each period (relative to all new users of the study DMARDs—low‐dose methotrexate and hydroxychloroquine—within each period) remained stable (*p* for trend = 0.93) and was similar in the hydroxychloroquine group (Table 1 and Table S7).

### 3.1 Study Outcomes

The primary outcome, death or a hospital visit with myelosuppression, sepsis, pneumotoxicity, or hepatotoxicity, occurred in 16 of 55 adults (29.1%) who started low‐dose methotrexate and in 29 of 407 adults (7.1%) who started hydroxychloroquine; the unadjusted RR was 4.08 (95% CI, 2.38 to 7.02), the unadjusted RD was 21.97% (95% CI, 9.7% to 34.2%), and the unadjusted NNH was 5 (95% CI, 3 to 11) (Table 2). The association between low‐dose methotrexate use and the primary outcome remained statistically significant after adjusting for baseline age, sex, and the total number of unique medications prescribed—aRR: 3.14 (95% CI, 1.75 to 5.63), aRD: 19.49% (95% CI, 7.53% to 31.44%), and aNNH: 6 (95% CI, 4 to 14).

**TABLE 2:** Risk of death or a hospital visit with myelosuppression, sepsis, pneumotoxicity, or hepatotoxicity in adults receiving maintenance dialysis within 90 days of starting a new prescription for low‐dose methotrexate vs. hydroxychloroquine^a^.

	Events, *n* (%)							
	Low‐dose methotrexate (*n* = 55)	Hydroxy‐chloroquine (*n* = 407)	Unadjusted RD, % (95% CI)	Unadjusted NNH (95% CI)	Unadjusted RR (95% CI)	Adjusted RD, % (95% CI)^b^	Adjusted NNH (95% CI)	Adjusted RR (95% CI)^b^
Primary outcomes								
Death or hospital visit with myelosuppression, sepsis, pneumotoxicity, or hepatotoxicity^c^	16 (29.1)	29 (7.1)	21.97 (9.70 to 34.23)	5 (3 to 11)	4.08 (2.38 to 7.02)	19.49 (7.53 to 31.44)	6 (4 to 14)	3.14 (1.75 to 5.63)
Secondary outcomes								
All‐cause hospitalization	23 (41.8)	92 (22.6)	19.21 (5.56 to 32.87)	6 (4 to 18)	1.85 (1.29 to 2.65)	18.94 (5.28 to 32.61)	6 (4 to 19)	1.79 (1.24 to 2.58)

Abbreviations: NNH, number needed to harm; RD, risk difference; RR, relative risk.

^a^ Reference group: Hydroxychloroquine.

^b^ Because of the small sample of methotrexate users, the regression analyses were adjusted for only 3 key risk factors—age (years), sex (male/female), and the number of unique drugs prescribed in the 120 days before cohort entry—to reduce the risk of overfitting. This approach aligns with the traditional recommendation of limiting the number of covariates relative to the number of outcome events (e.g., 10 events per variable).

^c^ The 90‐day risk of death or a hospital visit with myelosuppression, sepsis, pneumotoxicity, or hepatotoxicity. The components of the primary outcome are not mutually exclusive, and as such, some individuals experienced more than one of the components of the primary outcome. To comply with ICES privacy regulations to minimize the risk of identification, specific values of cells with ≤ 5 adults were suppressed. As a result, the components of the primary outcome are not presented in the table.

Low‐dose methotrexate use was also significantly associated with a higher 90‐day risk of all‐cause hospitalization (Table 2); however, further analysis of the individual components of the primary outcome was not possible due to the small number of events (death, myelosuppression, sepsis, pneumotoxicity, and hepatotoxicity). The overall event rate for each outcome component is shown in Table S8.

### 3.2 Post Hoc Analyses

The primary findings were supported by the post hoc analyses. (1) The risk estimates adjusted for predictors identified using a random forest algorithm were similar to those obtained in the primary analysis—aRR: 3.19 (95% CI, 1.78 to 5.72), aRD: 18.82% (95% CI, 7.05% to 30.60%), and aNNH: 6 (95% CI, 4 to 15). (2) The E‐values for the aRR and its lower confidence bound were 5.73 and 2.90, respectively, suggesting that substantial unmeasured confounding would be required to explain away the observed association (Supplementary Figure S2). (3) The main findings were supported by the absence of a positive association with a negative‐control outcome (a thyroid test during a hospital visit) (Supplementary Table S9). When adjusted for 55 baseline variables with standardized differences > 10%, the effect estimates remained consistent with those from the primary analysis (Table S10).

## 4 Discussion

In this population‐based cohort study of 462 adults receiving maintenance dialysis, those who started a prescription for low‐dose methotrexate (median dose 10 mg/week) vs. hydroxychloroquine experienced a significantly higher 90‐day risk of death or hospitalization with myelosuppression, sepsis, pneumotoxicity, or hepatotoxicity. Among patients receiving dialysis, approximately 1 in 3 who started low‐dose methotrexate experienced a serious adverse event or died within 90 days of starting the medication.

These findings confirm and extend evidence from 22 case reports linking low‐dose methotrexate use to toxicity and death in adults receiving dialysis (Table S2). Taken together, the findings from this study and others support current product monographs and prescribing guidelines that recommend against the use of low‐dose methotrexate in this population. Notably, the absolute number of adults prescribed low‐dose methotrexate has increased over time, despite existing recommendations to avoid it. While the study's primary aim was not to evaluate the risk–benefit profile of low‐dose methotrexate, our findings underscore the importance of careful consideration when initiating this medication in adults receiving dialysis.

This study has several strengths. This is the first population‐based study to assess the risk of serious adverse events following the start of low‐dose methotrexate compared with another DMARD in adults receiving dialysis. The study was conducted in the context of usual clinical care and included a representative sample of adults receiving dialysis in Ontario, Canada, where 90% of patients have universal prescription drug coverage. Results were supported by multiple sensitivity analyses. While it is well recognized that methotrexate should generally be avoided in patients receiving dialysis due to its toxicity, our study adds important real‐world, population‐based evidence that quantifies this risk. We used adjusted regression models that accounted for several strong risk factors to estimate the 90‐day risk of serious adverse events and death. By providing these adjusted risk estimates, our findings go beyond prior clinical warnings or anecdotal reports and offer concrete data to support safer prescribing and reinforce existing guidelines.

This study has some limitations. First, observational studies cannot definitively establish causality, and replication is warranted. Second, while prescription dispensing is accurately recorded in our databases, patient adherence is unknown. Third, we only captured adverse events leading to hospital visits; less severe events managed in outpatient settings were not captured, and therefore, the total morbidity of low‐dose methotrexate use in the dialysis population remains unknown. Fourth, the study did not assess the risk–benefit ratio of using low‐dose methotrexate. Fifth, because of the small sample size and limited number of events, we were unable to perform propensity score or high‐dimensional propensity score (hdPS) adjustment to balance the 95 baseline characteristics, including treatment indications. While hdPS is commonly used in large administrative datasets to empirically identify proxies for unmeasured confounders, its application in smaller cohorts—for example, our study with only 16 exposed patients who experienced the outcome—can lead to overfitting or model instability [30]. To address this, we prioritized model parsimony in our primary analysis and adjusted for three key, clinically relevant confounders—age, sex, and a proxy for polypharmacy—consistent with recommended events‐per‐variable guidelines. To further mitigate potential confounding, we conducted a post hoc random forest analysis to identify the most predictive covariates (exposure, polypharmacy, prescriber specialty) and incorporated these into the final regression models; results remained consistent with the primary findings. We also performed a post hoc sensitivity analysis adjusting for 55 baseline variables with standardized differences > 10%, yielding stable results. Additionally, a large E‐value and a negative‐control outcome analysis provided further reassurance that substantial unmeasured confounding would be needed to explain away the observed association. Despite these efforts, we acknowledge that some residual confounding may still be present. Sixth, we were unable to determine the specific clinical indications for low‐dose methotrexate use, as this information is not directly captured in administrative data. However, diagnostic codes prior to treatment initiation suggest that patients had autoimmune conditions such as rheumatoid arthritis, atopic dermatitis or eczema, psoriasis, systemic lupus erythematosus, and dermatomyositis (Table 1 and Table S7). This heterogeneity in underlying conditions may have contributed to differences in prognosis and treatment‐related risks, which we acknowledge as a limitation. Seventh, although we did not conduct a sensitivity analysis using an as‐treated approach, the short follow‐up period and the exclusion of patients receiving other DMARDs at cohort entry make it unlikely that treatment changes during follow‐up meaningfully influenced the results. Any crossover would likely bias effect estimates toward the null, suggesting that our findings are conservative. Eighth, while vascular access type (catheter vs. arteriovenous fistula) was not assessed, the low number of sepsis events (*n* = 17) within our composite primary outcome (Table S8) limits its potential as a confounder for the DMARD safety estimates. Ninth, we analyzed all‐cause mortality as an associated event rather than a definitively “drug‐related” outcome. Administrative databases do not provide access to coroner reports, autopsy results, or detailed bedside clinical narratives, so we could not establish direct causal relatedness for individual deaths. The 90‐day follow‐up window was chosen to capture acute events. Despite these limitations, the observed three‐fold higher risk with low‐dose methotrexate compared with hydroxychloroquine indicates a robust safety signal, unlikely to be explained solely by background dialysis mortality. Lastly, the low number of events for the components of the primary outcome precluded further analyses of these outcomes. In addition, the small number of patients who received low‐dose methotrexate limited the precision of our estimates, as reflected in the wide CIs. However, the association between low‐dose methotrexate use and the risk of serious adverse events and death remained statistically significant. This suggests that, despite limited precision, the observed association is unlikely to be due to chance. Moreover, the direction and consistency of the effect across multiple sensitivity analyses support the robustness and clinical relevance of the findings.

These findings inform clinical practice. (1) Clinicians should avoid prescribing low‐dose methotrexate to adults receiving dialysis when alternatives are available. (2) Efforts should be made to enhance physician awareness of safe prescribing practices for adults receiving dialysis and to improve communication between healthcare providers. (3) Computerized provider order entry systems should be updated to include dialysis‐specific medication alerts. (4) Product monographs and prescribing guidelines should be revised to incorporate these findings. Finally, regulatory agencies such as Health Canada and the US Food and Drug Administration should consider issuing warning labels to alert prescribers of the study findings. Furthermore, future research should investigate whether certain subgroups, such as patients on peritoneal dialysis or those with residual renal function, experience different risk profiles. Tailored prescribing recommendations or enhanced monitoring strategies may be warranted for these groups to further improve medication safety.

Among patients receiving dialysis, approximately 1 in 3 who started low‐dose methotrexate experienced a serious adverse event or died within 90 days of starting the medication. Our study highlights a significantly higher risk of harm after initiating low‐dose methotrexate compared with hydroxychloroquine. Until more data are available, low‐dose methotrexate should be avoided in adults receiving dialysis whenever possible, and alternative DMARDs should be considered.

## Funding

The research was conducted by members of the ICES Kidney, Dialysis and Transplantation team at the ICES Western facility, who are supported by a grant from the Canadian Institutes of Health Research (CIHR). This study was supported by ICES, which is funded by an annual grant from the Ontario Ministry of Health (MOH) and the Ministry of Long‐Term Care (MLTC). The study was completed at the ICES Western site, where core funding is provided by the Academic Medical Organization of Southwestern Ontario, the Schulich School of Medicine and Dentistry, Western University, and the Lawson Health Research Institute. This document used data adapted from the Statistics Canada Postal CodeOM Conversion File, which is based on data licensed from Canada Post Corporation, and/or data adapted from the Ontario Ministry of Health Postal Code Conversion File, which contains data copied under license from Canada Post Corporation and Statistics Canada. We thank IQVIA Solutions Canada Inc. for use of their Drug Information File. Parts of this material are based on data and information compiled and provided by the Canadian Institutes of Health Information (CIHI) and by the Ontario Ministry of Health (MOH). The analyses, conclusions, opinions, and statements expressed herein are solely those of the authors and do not reflect those of the funding or data sources; no endorsement is intended or should be inferred. The study sponsors had no role in the design and conduct of the study; the collection, management, analysis, and interpretation of the data; the preparation, review, or approval of the manuscript; or the decision to submit the manuscript for publication.

## Conflicts of Interest

The authors declare no conflicts of interest.

## Supporting information


**Figure S1:** Study design diagram comparing the use of low‐dose methotrexate versus hydroxychloroquine.
**Figure S2:** E‐value analysis to assess the extent of unmeasured confounding that would be required to negate the observed results.
**Table S1:** Literature search.
**Table S1a:** A literature search in Medline (1946 to April 7, 2025).
**Table 1b.** Literature search in Embase (1947 to April 7, 2025).
**Table 2** Summary of studies of methotrexate‐associated adverse events in patients receiving dialysis.
**Table S3:** The RECORD statement for pharmacoepidemiology (RECORD‐PE) checklist of items, extended from the STROBE and RECORD statements, which should be reported in non‐interventional pharmaco‐epidemiological studies using routinely collected health data.
**Table S4:** Coding definitions for demographic and comorbid conditions.
**Table S5:** Coding definitions for patients receiving maintenance dialysis.
**Table S6:** Operating characteristics of hospital diagnosis codes used to define the primary and secondary outcomes.
**Table S7:** Baseline characteristics^a^ of adults receiving maintenance dialysis newly prescribed low‐dose methotrexate versus those newly prescribed hydroxychloroquine (HCQ) in Ontario, Canada (1997–2020).
**Table S8:** Frequency of each component in the cohort.
**Table S9:** Risk of an outpatient or hospital visit with a thyroid disorder in adults receiving maintenance dialysis within 90 days of starting a new prescription for low‐dose methotrexate vs. hydroxychloroquine^a^.
**Table S10:** Risk of death or a hospital visit with myelosuppression, sepsis, pneumotoxicity, or hepatotoxicity in adults receiving maintenance dialysis within 90 days of starting a new prescription for low‐dose methotrexate vs. hydroxychloroquine after adjustment for 55 baseline characteristics ^a^.

## Data Availability

No additional data available because of governmental privacy regulations.
