# Supplementary material for: Risk of Serious Adverse Events and Death With Low‐Dose Methotrexate Versus Hydroxychloroquine in Adults Receiving Dialysis
Source: Semin Dial. 2026 May 12;39(4):168–76. doi: 10.1111/sdi.70035 (PMC13511332; doi:10.1111/sdi.70035)
Supplement: Supplementary file 1 [file SDI-39-168-s001.docx]

**Supplementary Material**

**Risk of Serious Adverse Events and Death with Low-Dose Methotrexate versus Hydroxychloroquine in Adults Receiving Dialysis**

**Supplementary tables**

**Supplementary Table S1**. Summary of studies of methotrexate-associated adverse events in patients receiving Dialysis.

**Supplementary Table S2.** Literature search.

**Supplementary Table S3.** The RECORD statement for pharmacoepidemiology (RECORD-PE) checklist of item, extended from the STROBE and RECORD statements, which should be reported in non-interventional pharmaco-epidemiological studies using routinely collected health data.

**Supplementary Table S4.** Coding definitions for demographic and comorbid conditions.

**Supplementary Table S5.** Coding definitions for patients receiving maintenance dialysis

**Supplementary Table S6.** Operating characteristics of hospital diagnosis codes used to define the primary and secondary outcomes

**Supplementary Table S7.** Baseline characteristics^a^ of adults receiving maintenance dialysis newly prescribed low-dose methotrexate versus those newly prescribed hydroxychloroquine (HCQ) in Ontario, Canada (1997–2020)

**Supplementary Table 8.** Frequency of each component in the cohort

**Supplementary Table 9.** Risk of an outpatient or hospital visit with a thyroid disorder in adults receiving maintenance dialysis within 90 days of starting a new prescription for low-dose methotrexate vs hydroxychloroquine **^a^**

**Supplementary Table 10.** Risk of death or a hospital visit with myelosuppression, sepsis, pneumotoxicity, or hepatotoxicity in adults receiving maintenance dialysis within 90 days of starting a new prescription for low-dose methotrexate vs hydroxychloroquine after adjustment for 55 baseline characteristics ^a^

**Supplementary Figures**

**Supplementary Figure S1.** Study design diagram comparing the use of low-dose methotrexate versus hydroxychloroquine

**Supplementary Figure S2.** E-value analysis to assess the extent of unmeasured confounding that would be required to negate the observed results

**Supplementary References**

**Supplementary Table S1.** Literature search

**Supplementary Table S1a.** A literature search in Medline (1946 to April 7, 2025)

| 1 | methotrexat*.ti,ab,kw. |
| --- | --- |
| 2 | methotrexat*.tw. /freq=2 |
| 3 | exp Methotrexate/ad, ae, po, tu, to [Administration & Dosage, Adverse Effects, Poisoning, Therapeutic Use, Toxicity] |
| 4 | 1 or 2 or 3 |
| 5* | exp Renal Dialysis/ or h?emodialy$.tw. or dialy$.ti. or peritoneal dialysis.mp. or dialysis patient$.tw. or ((end stage or endstage) adj (kidney or renal)).ti. or dialysis therapy.tw. or exp *Hemo filtration/ or *Renal Replacement Therapy/ or esrd.ti. or renal replacement.ti. or capd.tw. or ur?emic patient$.tw. or h?emo filt$.tw. or intradialy $.tw. or sevelamer.mp. or ur?emia.ti. or tenckhoff$.tw. or renal hyperparathyroidism.tw. or ccpd.tw. or nephrogenic systemic fibrosis.tw. or (((((kidney or renal) adj failure) or (chronic adj (kidney or renal))).tw. or Catheterization,Central Venous/ or Catheters, Indwelling/ or renal replacement.mp. or infection$.mp. or erythropoietin$.mp. or fistula$.tw. or hyperoxaluria.mp.) and dialysis.tw.) or (exp Renal Insuf ficiency/ and (Catheters, Indwelling/ or erythropoietin$.mp. or Catheterization, Central Venous/ or an?emi$.ti. or nephrogenic.tw. or amyloid$.mp.)) or ((chronic or end-stage).mp. and (renal replacement or azot?emia).tw.) or (((chronic adj (kidney or renal)) or ur?emi$ or ckd).ti. and (in flammation.tw. or erythropoietin$.mp. or renal osteodystrophy.mp. or hypertrophy.tw.)) or ((ur?emi$.ti. or *Uremia/) and (calci fication.tw. or hyperparathyroidism secondary.mp. or pruritus.mp. or secondary hyperparathyroidism.tw.)) or (((kidney or renal) adj transplant$) and candidates).tw. or (encapsulating.tw. and sclerosis.mp.) |
| 6 | exp "drug-related side effects and adverse reactions"/ or adverse.ti,ab,kf. or side effect?.ti,ab,kf. or adverse effects.fs. or exp drug overdose/ or overdos*.ti,ab,kf. or exp drug misuse/ or misus*.ti,ab,kf. or exp substance-related disorders/ or abus*.ti,ab,kf. or exp pregnancy/ or pregnan*.ti,ab,kf. or exp pregnancy complications/ or exp lactation/ or exp lactation disorders/ or exp breast feeding/ or (exp milk, human/ and exp secretion/) or exp fertility/ or exp infertility/ or exp reproduction/ or exp fetus/ or exp embryonic structures/ or terat*.ti,ab,kf. or drug efficacy.ti,ab,kf. or therapeutic efficacy.ti,ab,kf. or drug withdrawal.ti,ab,kf. or exp medication errors/ or exp death/ or death*.ti,ab,kf. or fatal*.ti,ab,kf. or exp drug interactions/ or exp carcinogens/ or carcinogen*.ti,ab,kf. or mutagen*.ti,ab,kf. or exp "off-label use"/ or exp occupational exposure/ or toxicity.fs. or toxic*.ti,ab,kf. or pharmacotox*.ti,ab,kf. or neurotox*.ti,ab,kf. or cardiotox*.ti,ab,kf. or nephrotox*.ti,ab,kf. or immunotox*.ti,ab,kf. or hepatotox*.ti,ab,kf. or cytotox*.ti,ab,kf. or immunocytotox*.ti,ab,kf. or intoxicat*.ti,ab,kf. or exp "congenital, hereditary, and neonatal diseases and abnormalities"/ or drug treatment failure.ti,ab,kf. or drug toxicity.ti,ab,kf. or exp case report/ or case report?.ti,ab,kf. or exp environmental exposure/ or treatment contraindication.ti,ab,kf. or exp contraindications, drug/ or exp "wounds and injuries"/ or suicid*.ti,ab,kf. or exp poisoning/ or poisoning.fs. or exp drug tolerance/ or exp treatment failure/ or exp drug resistance/ or exp substance-related disorders/ |
| 7 | 4 and 5 and 6 |
| 8 | limit 7 to (english language and humans) |

**Supplementary Table 1b.** Literature search in Embase (1947 to April 7, 2025)

| 1 | methotrexat*.ti,ab,kw. |
| --- | --- |
| 2 | methotrexat*.tw. /freq=2 |
| 3 | exp methotrexate/ae, ct, ad, cm, do, dt, to, im, iv, po, pv, sc, tm [Adverse Drug Reaction, Clinical Trial, Drug Administration, Drug Comparison, Drug Dose, Drug Therapy, Drug Toxicity, Intramuscular Drug Administration, Intravenous Drug Administration, Oral Drug Administration, Special Situation for Pharmacovigilance, Subcutaneous Drug Administration, Unexpected Outcome of Drug Treatment] |
| 4 | exp adverse drug reaction/ or adverse.ti,ab,kw. or side effect?.ti,ab,kw. or side effect.fs. or exp drug overdose/ or overdos*.ti,ab,kw. or exp drug misuse/ or misus*.ti,ab,kw. or exp drug abuse/ or exp substance abuse/ or abus*.ti,ab,kw. or exp pregnancy/ or pregnan*.ti,ab,kw. or exp pregnancy complications/ or exp lactation/ or exp breast feeding/ or (exp milk human/ and exp secretion/) or exp fertility/ or exp infertility/ or exp reproduction/ or exp fetus/ or exp embryo/ or terat*.ti,ab,kw. or exp drug efficacy/ or exp drug withdrawal/ or exp medication error/ or exp death/ or death*.ti,ab,kw. or fatal*.ti,ab,kw. or exp drug interaction/ or exp carcinogenicity/ or carcinogen*.ti,ab,kw. or mutagen*.ti,ab,kw. or exp 'off label drug use'/ or exp occupational exposure/ or exp toxicity/ or toxic*.ti,ab,kw. or pharmacotox*.ti,ab,kw. or neurotox*.ti,ab,kw. or cardiotox*.ti,ab,kw. or nephrotox*.ti,ab,kw. or immunotox*.ti,ab,kw. or hepatotox*.ti,ab,kw. or cytotox*.ti,ab,kw. or immunocytotox*.ti,ab,kw. or exp intoxication/ or exp congenital disorder/ or exp drug treatment failure/ or exp case report/ or case report?.ti,ab,kw. or exp environmental exposure/ or exp treatment contraindication/ or exp drug contraindication/ or exp injury/ or suicid*.ti,ab,kw. or exp poisoning/ or exp drug tolerance/ or exp treatment failure/ or exp drug resistance/ or exp substance-related disorders/ or drug resistance.fs. |
| 5* | exp *renal replacement therapy/ or h?emodialy$.tw. or peritoneal dialysis.mp. or dialy$.ti. or h?emodialysis patient$.mp. or dialysis patient$.tw. or exp *dialysis/ or ((end stage or endstage) adj (kidney or renal)).ti. or dialysis therapy.tw. or sevelamer.mp. or arti ficial kidney/ or h?emodia filt$.mp. or esrd.ti. or renal replacement.ti. or h? emo filt$.tw. or intradialy$.tw. or renal hyperparathyroidism.tw. or nephrogenic systemic fibrosis.tw. or ur?emic patient$.tw. or ((renal replacement.mp. or ((kidney or renal) adj failure).tw. or ur?emia.tw. or catheter$.mp. or erythropoietin$.mp. or fistula $.mp. or *chronic kidney failure/ or hyperkalemia.mp. or hyperoxaluria.tw. or exp blood vessel calci fication/ or anuria.mp.) and (dialys$ or h?emodialys$).mp.) or (((chronic adj (kidney or renal)) or ur?emi$ or ckd).ti. and (in flammation.tw. or renal osteodystrophy.mp. or hypertrophy.tw.)) or (((kidney or renal) adj transplant$).tw. and exp *urinary tract disease/ and (candidates or wait list$).tw.) |
| 6 | 1 or 2 or 3 |
| 7 | 4 and 5 and 6 |
| 8 | limit 7 to (human and english language) |

**Supplementary Table 2** Summary of studies of methotrexate-associated adverse events in patients receiving Dialysis

| Author/year | Design | Age^a^ | Sex | Type of Dialysis | Indication for LD-MTX use | Dose (mg/week)^a^ | Type of toxicity |
| --- | --- | --- | --- | --- | --- | --- | --- |
| Shively 2020^1^ | Case report | 77 | M | PD | RA | 7.5 | stomatitis, pancytopenia, mild hepatotoxicity |
| Basile 2002 ^2^ | Case report | 74 | F | HD | RA | 5 | GI ulcerations, severe pancytopenia |
| Boulanger 2001^3^ | Case report | 60 | F | HD | RA | 5 | pancytopenia, buccal ulcerations |
| Chatham ^4^ 2000 | Case report | 49 | M | HD | Myositis | IV weekly, no information on dose | GI ulcerations, febrile neutropenia and anemia |
|  | Case report | 52 | M | HD | Myositis | IM 5 | sepsis with pneumonia in the setting of neutropenia |
|  | Case report | 61 | F | HD | Psoriasis with arthropathy | IM 2.5 and 5 | fever and pancytopenia |
| Seneschal 2007 ^5^ | Case report | 76 | M | HD | Bullous pemphigoid | 5 | pancytopenia, pneumonia and sepsis |
| Aristizabal-Alzate 2018 ^6^ | Case report | 68 | M | PD | Vasculitis | 7.5 | Fever, pancytopenia, septic shock, skin and mucous membranes lesions |
| Chess 2004 ^7^ | Case report | 64 | M | PD | Psoriasis | cumulative of 35mg | neutropenia with fever |
| Cheung 2009 ^8^ | Case report | 56 | M | HD | Psoriasis with arthropathy | 2.5 | Pancytopenia and fever |
| Ellman 1990 ^9^ | Case report | 52 | F | PD | RA | 2.5 | ulcerations, neutropenia and thrombocytopenia |
|  | Case report | 47 | F | HD | Scleroderma and polymyositis | 2.5 | pneumonitis and neutropenia |
| Liu 2015 ^10^ | Case report | 48 | M | HD | Bullous pemphigoid | 10 | acute interstitial pneumonitis and pancytopenia |
| Liu 2014 ^11^ | Case report | 61 | F | PD | Intractable chronic eczema | 7.5 | fever, mucositis, pancytopenia |
| Mima 2017 ^12^ | Case report | 46 | M | HD | RA | No information on dose | pneumatosis intestinalis, myelosuppression |
| Sun 2006 ^13^ | Case report | 33 | F | PD | SLE | 5 for the first two weeks and then 7.5 mg for two weeks | pancytopenia and leukemoid reaction |
| Boey 2006 ^14^ | Case report | 66 | M | HD | Psoriatic arthritis | 5 | fever, mucositis, pancytopenia, liver toxicity |
| Yang 2006 ^15^ | Case report | 55 | F | HD | RA | 7.5 | pancytopenia, fever and ulcerations |
| Diskin 2006 ^16^ | Case report | 60 | M | PD | Bilateral symmetrical arthritis | SC 10 | Ulcerations and pancytopenia |
| Abdullah 2023^17^ | Case report | 49 | F | PD | RA | 10 | painful  mouth ulcers, fever, and menorrhagia, pancytopenia |
| Samantha  2022^18^ | Case report | 67 | M | PD | Psoriasis with psoriatic arthritis | 10 | abdominal pain, diarrhea, rash, and mucositis, pancytopenia |
| The Office of the Chief Coroner for Ontario , Canada 2018^19^ | Case report | 48 | M | HD | Sarcoidosis | 10mg in first week and 15 mg in the second week orally | Oral thrush, diarrhea, pancytopenia |

Abbreviations: M, Male; F, Female; PD, Peritoneal Dialysis; HD, hemodialysis; RA, Rheumatoid arthritis; GI, Gastrointestinal; SLE, Systemic lupus erythematosus; LD-MTX, low-dose methotrexate.

^a^The median [IQR] age and weekly dose were 58 (49 to 65.5) years and 7.5 (5 to 8.75) mg in these case reports when the information was available.

**Supplementary Table 3.** The RECORD statement for pharmacoepidemiology (RECORD-PE) checklist of items, extended from the STROBE and RECORD statements, which should be reported in non-interventional pharmaco-epidemiological studies using routinely collected health data

| **Item No** | **STROBE items** | **RECORD items** | **RECORD-PE items** | **Page No** |
| --- | --- | --- | --- | --- |
| **Title and abstract** | | | | |
| 1 | (a) Indicate the study’s design with a commonly used term in the title or the abstract.  (b) Provide in the abstract an informative and balanced summary of what was done and what was found. | 1.1: The type of data used should be specified in the title or abstract. When possible, the name of the databases used should be included.  1.2: If applicable, the geographical region and timeframe within which the study took place should be reported in the title or abstract.  1.3: If linkage between databases was conducted for the study, this should be clearly stated in the title or abstract. | — | 1-6 |
| **Introduction** | | | | |
| Background rationale | | | | |
| 2 | Explain the scientific background and rationale for the investigation being reported. | — | — | 4,5 |
| Objectives | | | | |
| 3 | State specific objectives, including any prespecified hypotheses. | — | — | 6 |
| **Methods** | | | | |
| Study design | | | | |
| 4 | Present key elements of study design early in the paper. | — | 4.a: Include details of the specific study design (and its features) and report the use of multiple designs if used.  4.b: The use of a diagram(s) is recommended to illustrate key aspects of the study design(s), including exposure, washout, lag and observation periods, and covariate definitions as relevant. | 6,7  Supplementary Figure S1 |
| Setting | | | | |
| 5 | Describe the setting, locations, and relevant dates, including periods of recruitment, exposure, follow-up, and data collection. | — | — | 6-9 |
| Participants | | | | |
| 6 | (a) Cohort study—give the eligibility criteria, and the sources and methods of selection of participants. Describe methods of follow-up. Case-control study—give the eligibility criteria, and the sources and methods of case ascertainment and control selection. Give the rationale for the choice of cases and controls. Cross-sectional study—give the eligibility criteria and the sources and methods of selection of participants.  (b) Cohort study—for matched studies, give matching criteria and number of exposed and unexposed. Case-control study—for matched studies, give matching criteria and the number of controls per case. | 6.1: The methods of study population selection (such as codes or algorithms used to identify participants) should be listed in detail. If this is not possible, an explanation should be provided.  6.2: Any validation studies of the codes or algorithms used to select the population should be referenced. If validation was conducted for this study and not published elsewhere, detailed methods and results should be provided.  6.3: If the study involved linkage of databases, consider use of a flow diagram or other graphical display to demonstrate the data linkage process, including the number of individuals with linked data at each stage. | 6.1.a: Describe the study entry criteria and the order in which these criteria were applied to identify the study population. Specify whether only users with a specific indication were included and whether patients were allowed to enter the study population once or if multiple entries were permitted. See explanatory document for guidance related to matched designs. | 7,8 |
| Variables | | | | |
| 7 | Clearly define all outcomes, exposures, predictors, potential confounders, and effect modifiers. Give diagnostic criteria, if applicable. | 7.1: A complete list of codes and algorithms used to classify exposures, outcomes, confounders, and effect modifiers should be provided. If these cannot be reported, an explanation should be provided. | 7.1.a: Describe how the drug exposure definition was developed.  7.1.b: Specify the data sources from which drug exposure information for individuals was obtained.  7.1.c: Describe the time window(s) during which an individual is considered exposed to the drug(s). The rationale for selecting a particular time window should be provided. The extent of potential left truncation or left censoring should be specified.  7.1.d: Justify how events are attributed to current, prior, ever, or cumulative drug exposure.  7.1.e: When examining drug dose and risk attribution, describe how current, historical or time on therapy are considered.  7.1.f: Use of any comparator groups should be outlined and justified.  7.1.g: Outline the approach used to handle individuals with more than one relevant drug exposure during the study period. | 7-9  Table 1  Supplementary Table S7 |
| Data sources/measurement | | | | |
| 8 | For each variable of interest, give sources of data and details of methods of assessment (measurement). Describe comparability of assessment methods if there is more than one group. | — | 8.a: Describe the healthcare system and mechanisms for generating the drug exposure records. Specify the care setting in which the drug(s) of interest was prescribed. | 6,7 |
| Bias | | | | |
| 9 | Describe any efforts to address potential sources of bias. | — | — | 7-9 |
| Study size | | | | |
| 10 | Explain how the study size was arrived at. | — | — | 9  Figure 1 |
| Quantitative variables | | | | |
| 11 | Explain how quantitative variables were handled in the analyses. If applicable, describe which groupings were chosen, and why. | — | — | 10-12 |
| Statistical methods | | | | |
| 12 | (a) Describe all statistical methods, including those used to control for confounding.  (b) Describe any methods used to examine subgroups and interactions.  (c) Explain how missing data were addressed.  (d) Cohort study—if applicable, explain how loss to follow-up was addressed. Case-control study—if applicable, explain how matching of cases and controls was addressed. Cross sectional study—if applicable, describe analytical methods taking account of sampling strategy.  (e) Describe any sensitivity analyses. | — | 12.1.a: Describe the methods used to evaluate whether the assumptions have been met.  12.1.b: Describe and justify the use of multiple designs, design features, or analytical approaches. | 7-9 |
| Data access and cleaning methods | | | | |
| 12 | — | 12.1: Authors should describe the extent to which the investigators had access to the database population used to create the study population.  12.2: Authors should provide information on the data cleaning methods used in the study. | — | 5,6 |
| Linkage | | | | |
| 12 | — | 12.3: State whether the study included person level, institutional level, or other data linkage across two or more databases. The methods of linkage and methods of linkage quality evaluation should be provided. | — | N/A |
| **Results** | | | | |
| Participants | | | | |
| 13 | (a) Report the numbers of individuals at each stage of the study (eg, numbers potentially eligible, examined for eligibility, confirmed eligible, included in the study, completing follow-up, and analysed).  (b) Give reasons for non-participation at each stage.  (c) Consider use of a flow diagram. | 13.1: Describe in detail the selection of the individuals included in the study (that is, study population selection) including filtering based on data quality, data availability, and linkage. The selection of included individuals can be described in the text or by means of the study flow diagram. | — | 9,10  Figure 1 |
| Descriptive data | | | | |
| 14 | (a) Give characteristics of study participants (eg, demographic, clinical, social) and information on exposures and potential confounders.  (b) Indicate the number of participants with missing data for each variable of interest.  (c) Cohort study—summarise follow-up time (eg, average and total amount). | — | — | 9,10  Table 1,  SupplementaryTable 7 |
| Outcome data | | | | |
| 15 | Cohort study—report numbers of outcome events or summary measures over time. Case-control study—report numbers in each exposure category, or summary measures of exposure. Cross sectional study—report numbers of outcome events or summary measures. | — | — | 9-11  Table2, |
| Main results | | | | |
| 16 | (a) Give unadjusted estimates and, if applicable, confounder adjusted estimates and their precision (eg, 95% confidence intervals). Make clear which confounders were adjusted for and why they were included.  (b) Report category boundaries when continuous variables are categorised.  (c) If relevant, consider translating estimates of relative risk into absolute risk for a meaningful time period. | — | — | 9-11  Table 2 |
| Other analyses | | | | |
| 17 | Report other analyses done—eg, analyses of subgroups and interactions, and sensitivity analyses. | — | — | 9-11  Table 2 |
| **Discussion** | | | | |
| Key results | | | | |
| 18 | Summarise key results with reference to study objectives. | — | — | 11,12 |
| Limitations | | | | |
| 19 | Discuss limitations of the study, taking into account sources of potential bias or imprecision. Discuss both direction and magnitude of any potential bias. | 19.1: Discuss the implications of using data that were not created or collected to answer the specific research question(s). Include discussion of misclassification bias, unmeasured confounding, missing data, and changing eligibility over time, as they pertain to the study being reported | 19.1.a: Describe the degree to which the chosen database(s) adequately captures the drug exposure(s) of interest. | 12-14 |
| Interpretation | | | | |
| 20 | Give a cautious overall interpretation of results considering objectives, limitations, multiplicity of analyses, results from similar studies, and other relevant evidence. | — | 20.a: Discuss the potential for confounding by indication, contraindication or disease severity or selection bias (healthy adherer/sick stopper) as alternative explanations for the study findings when relevant. [A: Original text indicated this item was RECORD (ie, not RECORD-PE)?] | 12-14 |
| Generalisability | | | | |
| 21 | Discuss the generalisability (external validity) of the study results. | — | — | 12-14 |
| **Other information** | | | | |
| Funding | | | | |
| 22 | Give the source of funding and the role of the funders for the present study and, if applicable, for the original study on which the present article is based. | — | — | 14 |
| Accessibility of protocol, raw data, and programming code | | | | |
| 22 | — | 22.1: Authors should provide information on how to access any Supplementaryinformation such as the study protocol, raw data, or programming code. | — | N/A |

**Supplementary Table S4.** Coding definitions for demographic and comorbid conditions

| **Characteristic** | **Database** | **Codes** |
| --- | --- | --- |
| **Demographics** |  |  |
| Age | RPDB |  |
| Sex | RPDB |  |
| Residential status – Long-term care | ODB | LTC |
| Location of residence – Rural | Statistics Canada |  |
| Socioeconomic Status – Neighbourhood Income Quintile | Statistics Canada |  |
| Year of cohort entry | ODB |  |
| LHIN^a^ | RPDB |  |
| Prescriber information | ODB |  |
| **Comorbidities (5 years prior to cohort entry date)** |  |  |
| Acute kidney injury | CIHI-DAD | ICD-9: “584”  ICD-10: “N17” |
| Alcohol misuse | CIHI-DAD | ICD-9: "303", "3050"  ICD-10: "E24", "E512", "F10", "G312", "G621", "G721", "I426", "K292", "K70", "K860", "T510", "X45", "X65", "Y15", "Y573", "Z502", "Z714", "Z721" |
|  | OHIP | OHIP DX: "303" |
| Angina | CIHI-DAD | ICD-9: "413"  ICD-10: "I20", "I23" |
|  | OHIP | OHIP DX: "413" |
| Atrial fibrillation/flutter | CIHI-DAD | ICD9: "4273"  ICD10: "I48" |
| Bipolar disorder | CIHI-DAD | ICD-9: "2960", "2961", "2964", "2965", "2966", "2967", "2968"  ICD-10: "F300", "F301", "F302", "F308", "F309", "F310", "F311", "F312", "F313", "F314", "F315", "F316", "F317", "F318", "F319" |
|  | OHIP | OHIP DX: "296"  OHIP FEE: "Q020" |
|  | OHMRS | DSM-IV: "29600", "29601", "29602", "29603", "29604", "29605", "29606", "29640", "29641", "29642", "29643", "29644", "29645", "29646", "29650", "29651", "29652", "29653", "29654", "29655", "29656", "29660", "29661", "29662", "29663", "29664", "29665", "29666", "29670", "29680", "29689" |
| Chronic liver disease | CIHI-DAD | ICD-9: "4561", "4562", "070", "5722", "5723", "5724", "5728", "573", "7824", "V026", "2750", "2751", "7891", "7895", "571"  ICD-10: "B16", "B17", "B18", "B19", "I85", "R17", "R18", "R160", "R162", "B942", "Z225", "E831", "E830", "K70", "K713", "K714", "K715", "K717", "K721", "K729", "K73", "K74", "K753", "K754", "K758", "K759", "K76", "K77" |
|  | OHIP | OHIP DX: "571", "573", "070"  OHIP FEE: "Z551", "Z554" |
| Chronic obstructive pulmonary disease | CIHI-DAD | ICD9: "491", "492", "496"  ICD10: "J41", "J43", "J44" |
| Cirrhosis | CIHI-DAD | ICD-9: “5711”, “5712”, “5713”, “5715”, “5716”, “5718”, “5719”  ICD-10: “K702”, “K703”, “K704, “K709, “K740”, “K741”, “K742”, “K743”, “K744”, “K745”, “K746” |
|  | OHIP | OHIP DX: “571”, “573” |
| Coronary artery disease (minus angina) | CIHI-DAD | ICD9: "412", "410", "411"  ICD10: "I21", "I22", "Z955", "T822"  CCI: "1IJ50", "1IJ76"  CCP: "4801", "4802", "4803", "4804", "4805", "481", "482", "483" |
|  | OHIP | OHIP Fee: "R741", "R742", "R743", "G298", "E646", "E651", "E652", "E654", "E655", "Z434", "Z448"  OHIPDx: "410", "412" |
| Dementia | CIHI-DAD | ICD-9: "2900", "2901", "2903", "2904", "2908", "2909", "2948", "2949", "3310", "3311", "3312", "2941", "797"  ICD-10: "F065", "F066", "F068", "F069", "F09", "F00", "F01", "F02", "F03", "F051", "G30", "G31", "R54" |
|  | OHIP | OHIP DX: "290", "331", "797" |
|  | OHMRS | DSM-IV: "29040", "29041", "29042", "29043", "29120", "29282", "29410", "29411", "29480", "78090" |
| Falls | CIHI-DAD | ICD-9: “E880”, “E881”, “E882”, “E883”, “E884”, “E885”, “E886”, “E887”, “E888”  ICD-10: “W00”, “W01”, “W02”, “W03”, “W04”, “W05”, “W06”, “W07”, “W08”, “W09”, “W10”, “W11”, “W12”, “W13”, “W14”, “W15”, “W16”, “W17”, “W18”, “W19” |
|  | OHIP | OHIP DX: "627" |
| Diabetes | ODB | Insulins, oral antihyperglycemic agents |
| Anemia | CIHI-DAD | ICD-9: "2800", "2801", "2808", "2809", "2810", "2811", "2812", "2813", "2814", "2818", "2819", "2820", "2821", "2822", "2823","2824", "2825", "28260", "28261", "28262", "28263", "28269", "2827", "2828", "2829", "2830", "2831", "2832", "2839", "2840", "2848", "2849", "2850", "2851", "2852", "2858","2859"  ICD-10: "D50”, "D51", "D52", "D53", "D55", "D56", "D570", "D571", "D58", "D59", "D60", "D61", "D62”, "D63”, "D64" |
|  | OHIP | OHIP DX: "280", "281", "282", "283", "284", "285" |
| Glaucoma | CIHI-DAD | ICD-9: "V801", "36500", "36501", "36502", "36503", "36504", "36510", "36511", "36512", "36513", "36514", "36515", "36520", "36521", "36522", "36523", "36524", "36531", "36532", "36541", "36542", "36543", "36544", "36551", "36552", "36559", "36560", "36561", "36562", "36563", "36564", "36565", "36581", "36582", "36589", "3659"  ICD-10: "H40"  CCI: "CZ28XK"  CCP: "0926" |
|  | OHIP | OHIP FEE: "E123", "E133", "E214", "E983", "E984", "G819", "G820" |
| Major hemorrhage | CIHI-DAD | ICD-9: "430", "431", "432", "5307", "5310", "5312", "5314", "5316", "5320", "5322", "5324", "5326", "5330", "5332", "5334", "5336", "5340", "5342", "5344", "5346", "5780", "5781", "5693", "5789"  ICD-10: "I600", "I601", "I602", "I603", "I604", "I605", "I606", "I607", "I609", "I61", "162", "I850", "I9820", "I983", "K2210", "K2211", "K2212", "K2214", "K2216", "K226", "K228", "K250", "K252", "K254", "K256", "K260", "K262", "K264", "K266", "K270", "K272", "K274", "K276", "K280", "K282", "K284", "K286", "K290", "K2921", "K2941", "K2951", "K2961", "K2971", "K2981", "K2991", "K3180", "K31811", "K3182", "K6380", "K920", "K921", "K5520", "K625", "K922" |
| Heart failure | CIHI-DAD | ICD-9: "425", "5184", "514", "428"  ICD-10: "I500", "I501", "I509", "I255", "J81"  CCP: "4961", "4962", "4963", "4964"  CCI: "1HP53", "1HP55", "1HZ53GRFR", "1HZ53LAFR", "1HZ53SYFR" |
|  | OHIP | OHIP FEE: "R701", "R702", "Z429"  OHIP DX: "428" |
| Hypertension | ODB | Antihypertensive agents |
| Hypokalemia | CIHI-DAD | ICD-9: “2768”  ICD-10: “E876” |
| Hyponatremia | CIHI-DAD | ICP-9: “2761”  ICD-10: “E871” |
| Hypothyroidism | CIHI-DAD | ICD-9: “243”, “2440”, “2441”, “2442”, “2443”, “2448”, “2449”  ICD-10: “E030”, “E031”, “E032”, “E033”, “E034”, “E035”, “E038”, “E039”, “E890” |
|  | OHIP | OHIP DX: “243”, “244” |
| Hypercalcemia | CIHI-DAD | ICD-9: “2754”  ICD-10: “E835” |
| Migraine | CIHI-DAD | ICD-9: “3460”, “3461”, “3462”, “3468”, “3469”  ICD-10: “G430”, “G431”, “G432”, “G433”, “G438”, “G439” |
|  | OHIP | OHIP DX: “346” |
| Acute myocardial infarction | CIHI-DAD | ICD9: "410"  ICD10: "I21", "I22" |
| Obesity | CIHI-DAD | ICD-9: "2780"  ICD-10: "E660", "E661", "E662", "E668", "E669" |
|  | OHIP | OHIP DX: "278" |
| Parkinson disease | CIHI-DAD | ICD9: "332"  ICD10: "G20", "F023” |
|  | OHIP | OHIP DX: "332" |
| Peripheral vascular disease | CIHI-DAD | ICD-9: "4402", "4408", "4409", "5571", "4439", "444"  ICD-10: "I700", "I702", "I708", "I709", "I731", "I738", "I739", "K551"  CCP: "5125", "5129", "5014", "5016", "5018", "5028", "5038", "5126", "5159"  CCI: "1KA76", "1KA50", "1KE76", "1KG26", "1KG50", "1KG57", "1KG76MI", "1KG87", "1IA87LA", "1IB87LA", "1IC87LA", "1ID87", "1KA87LA", "1KE57" |
|  | OHIP | OHIP FEE: "R787", "R780", "R797", "R804", "R809", "R875", "R815", "R936", "R783", "R784","R785", "E626", "R814", "R786", "R937", "R860", "R861", "R855", "R856", "R933", "R934", "R791", "E672", "R794", "R813", "R867", "E649" |
| Schizophrenia | CIHI-DAD | ICD-9: "2950", "2951", "2952", "2953", "2954", "2955", "2956", "2957", "2958", "2959", "2970", "2971", "2972", "2973", "2978", "2979", "2980", "2981", "2983", "2984", "2988", "2989"  ICD-10: "F060", "F062", "F105", "F107", "F115", "F117", "F125", "F127", "F135", "F137", "F145", "F147", "F155", "F157", "F165", "F167", "F175", "F177", "F185", "F187", "F195", "F197", "F200", "F201", "F202", "F203", "F204", "F205", "F206", "F208", "F209", "F220", "F228", "F229", "F230", "F231", "F232", "F233", "F238", "F239", "F24", "F250", "F251", "F252", "F258", "F259", "F28", "F29" |
|  | OHIP | OHIP DX: "291", "292", "295", "297", "298"  OHIP FEE: "Q021" |
|  | OHMRS | DSM-IV: "29130", "29150", "29211", "29212", "29381", "29382", "29510", "29520", "29530", "29540", "29560", "29570", "29590", "29710", "29730", "29880", "29890" |
| All stroke (including transient ischemic attack) | CIHI-DAD | ICD-9: "430", "431", "432", "434", "435", "436", "3623"  ICD-10: "I62", "I630", "I631", "I632", "I633", "I634", "I635", "I638", "I639", "I64", "H341", "I600", "I601", "I602", "I603", "I604", "I605", "I606", "I607", "I609", "I61", "G450", "G451", "G452", "G453", "G458", "G459", "H340" |
| Unipolar depression and/or anxiety disorder | CIHI-DAD | ICD-9: "2962", "2963", "3000", "3002", "3003", "3004", "3091", "311"  ICD-10: "F063", "F064", "F320", "F321", "F322", "F323", "F328", "F329", "F330", "F331", "F332", "F333", "F334", "F338", "F339", "F341", "F400", "F401", "F402", "F408", "F409", "F410", "F411", "F412", "F413", "F418", "F419", "F420", "F421", "F422", "F428", "F429", "F430", "F431" |
|  | OHIP | OHIP-DX: "311" |
|  | OHMRS | DSM-IV: "29189", "29284", "29289", "29383", "29384", "29620", "29621", "29622", "29623", "29624", "29625", "29626", "29630", "29631", "29632", "29633", "29634", "29635", "29636", "30000", "30001", "30002", "30021", "30022", "30023", "30029", "30030", "30040", "30113" |
| Ventricular arrhythmia | CIHI-DAD | ICD9: "4274"  ICD-10: "I4900", "I472" |
| Rheumatoid arthritis | CIHI-DAD | ICD-9: "714"  ICD-10: "M05", "M06" |
|  | OHIP | OHIP DX: "714" |
| Syncope | CIHI-DAD | ICD-9: "7802"  ICD-10: "R55" |
| Dermatomyositis | CIHI-DAD | ICD-9: 7103  ICD10: M330, M331 |
|  | OHIP | OHIP DX:710 |
| Systemic sclerosis | CIHI-DAD | ICD-9: 7010, 7101,6958  ICD10:L940, L941, M342, M340, M341, M348, M349 |
|  | OHIP | OHIP DX:701 |
| Atopic dermatitis or eczema | CIHI-DAD | ICD-9: 6918, 0540  ICD10: L208, L209, B000 |
|  | OHIP | OHIP DX: 691 |
| Benign prostatic hyperplasia | CIHI-DAD | ICD-9: "600"  ICD-10: "N40" |
|  | OHIP | OHIP DX: "600" |
| Prostatitis | CIHI-DAD | ICD-9: "6010", "6011", "6012"  ICD-10: "N410", "N411", "N412" |
|  | OHIP | OHIP DX: "601" |
| Hypotension | CIHI-DAD | ICD-9: "458"  ICD-10: "I95" |
| Sarcoidosis | CIHI-DAD | ICD-9: 135, 3574, 7104  ICD10: D860, D861, D862, D863, D868, D869, G532, M633 |
|  | OHIP | OHIP DX: “135” |
| Ulcerative colitis (UC) | CIHI-DAD | ICD-9: "556"  ICD-10: "K51" |
|  | OHIP | OHIP: "556" |
| Psoriasis | CIHI-DAD | ICD-9: 6960, 6961, 6962, 6963,6968, 6969, 6964  ICD10: L400, L401, L404, L405, L408, L409, L413, L414, L418, L415, L419, M070, M072, M073, M090 |
|  | OHIP | OHIP DX: 696 |
| Acute urinary retention | CIHI-DAD | ICD-9: "7882"  ICD-10: "R33" |
| Nystagmus and other irregular eye movements | CIHI-DAD | ICD-9: "3795"  ICD-10: "H55" |
|  | OHIP | OHIP DX: "379" |
| Fracture | CIHI-DAD | ICD9: "8200", "8201", "8208", "8209", "8202", "8203", "8210", "8211", "S723", "813", "812", "8056", "8057", "808"  ICD10: "S720", "S721", "S722", "S52", "S422", "S321", "S322", "S324", "S323", "S325", "S327", "S328"  CCP: "9104", "9124", "9054", "9114", "9134", "935", "936", "9104", "9124", "9054", "9114", "9134"  , "9101", "9121", "9141", "9111", "9131", "9052"  CCI: "1VA73", "1VC73", "1VA74", "1VA53", "1VC74", "1VA80", "1VC73", "1VC74", "1VC03", "1VC80", "1TV73", "1TV74", "1TV03" |
|  | OHIP | OHIP FEE: "F095", "F096", "F097", "Z211", F014", "F022", "F023", "F025", "F026", "F028", "F030", "F032", "F033", "F046", "F024", "F027", "F031", "Z203", |
| Macular degeneration | CIHI-DAD | ICD-9: “362”  ICD-10: “H35” |
|  | OHIP | OHIP FEE: “E154”, “E125”, “E126”, “E147” |
| Gastroesophageal reflux disease | CIHI-DAD | ICD-9: "530", "531", "532", "533", "534", "535", "536", "537", "538", "539"  ICD-10: "K21" |
|  | OHIP | OHIP DX: "530" , "531" , "532" , "533" , "534" , "535" , "536" , "537" , "538" , "539" |
| Osteoarthritis | CIHI-DAD | ICD9: "715"  ICD10: "M15", "M16", "M17", "M18", "M19", "M47" |
| Prostate cancer | CIHI-DAD | ICD-9: "185", "2334"  ICD-10: "C61", "D075" |
|  | OHIP | OHIP DX: "185" |
| Transient ischemic stroke | CIHI-DAD | ICD9: "435"  ICD10: "H340", "G450", "G451", "G452", "G453", "G458", "G459" |
| Gout | CIHI-DAD | ICD9: "274"  ICD10: "M10" |
|  | OHIP | OHIP dx: "274" |
| **Medication use (120 days prior to cohort entry date)** |  |  |
| All | ODB |  |
| **Healthcare use (1 year prior to cohort entry date)** |  |  |
| GP/FP visits | OHIP, IPDB | Mainspecialty = “GP/FP” or “F.P./Emergency Medicine” |
| Nephrologist visits | OHIP, IPDB | Mainspecialty = NEUROLOGY |
| Psychiatrist visits | OHIP, IPDB | Mainspecialty = PSYCHIATRY |
| Number of Hospitalizations | CIHI-DAD | “admdate” |
| Number of Emergency department visits | NACRS | “regdate” |
| Number of serum creatinine tests | OHIP | OHIP FEE: "L065", "L067", "L068" |
| TSH test | OHIP | OHIP FEE: "G016", "G399", "L341" |
| At home physician service | OHIP | OHIP FEE: "A901", "B960", "B961", "B962", "B963", "B964", "B966", "B990", "B992", "B993", "B994", "B996", "B997", "B998" |
| Bone mineral density test | OHIP | OHIP FEE: "J654", "J688", "J854", "J888", "X149", "X152", "X153", "X155", "Y654", "Y688", "Y854", "Y888" |
| Cardiac catheterization | CIHI-DAD | CCP: "4995", "4996", "4997"  CCI: "3IJ30GP", "3HZ30GP", "2HZ24GPKJ", "2HZ24GPKL", "2HZ24GPKM", "2HZ24GPXJ", "2HZ28GPPL", "2HZ71GP" |
|  | OHIP | OHIP FEE: "G296", "G297", "G299", "G300", "G301", "G304", "G305", "G306" |
| Cardiac stress test | CIHI-DAD | CCP: "0341", "0342", "0343", "0344", "0605"  CCI: "2HZ08", "3IP70” |
|  | OHIP | OHIP FEE: "G315", "G174", "G111", "G112", "G319", "G582", "G583", "G584", "J604", "J606", "J607", "J608", "J609", "J611", "J612", "J613", "J667", "J807", "J808", "J809", "J804", "J811", "J812", "J813", "J867", "J666", "J866" |
| Carotid ultrasound | CIHI-DAD | CCP: "0281"  CCI: "3JE30", "3JG30" |
|  | OHIP | OHIP FEE: "J201", "J501", "J189", "J489', "J190", "J191", "J490", "J491", "J492" |
| Chest X-ray | OHIP | OHIP FEE: "X090", "X091", "X092", "X195" |
| Cataract surgery | OHIP | OHIP FEE: "E140" |
| Cervical cancer screening | OHIP | OHIP FEE: "E430", "G365", "G394", "L713", "L812” |
| Colorectal cancer screening | OHIP | OHIP FEE: "G004", "L179", "L181", "Q043", "Q152", "X112", "X113", "Z535", "Z536", "Z555", "Z580" |
| Cholesterol test (total cholesterol, HDL) | OHIP | OHIP FEE: "G001", "G013", "L117", "Q183" |
| CT Abdomen | OHIP | OHIP FEE: "X126", "X409", "X410" |
| CT Head | OHIP | OHIP FEE: "X188", "X400", "X401", "X402", "X405", "X408" |
| CT Pelvis | OHIP | OHIP FEE: "X231", "X232", "X233" |
| CT Spine | OHIP | OHIP FEE: "X128", "X415", "X416" |
| CT Thorax | OHIP | OHIP FEE: "X125", "X406", "X407" |
| Echocardiography | CIHI-DAD | CCP: "0282"  CCI: "3IP30" |
|  | OHIP | OHIP FEE: "G560", "G561", "G562", "G566", "G567", "G568", "G570", "G571", "G572", "G574", "G575", "G576", "G577", "G578", "G579", "G580", "G581" |
| Flu shot | OHIP | OHIP FEE: "G590", "G591" |
| Cystoscopy |  |  |
| Hearing test | OHIP | OHIP FEE: "G153", "G154", "G440", "G441", "G442", "G443", "G448", "G450", "G451", "G452", "G525", "G526", "G529", "G530", "G533", "G815", "G816" |
| Mammography | OHIP | OHIP FEE: "X172", "X178", "X184", "X185", "X201" |
| Prostate specific antigen (psa) test | OHIP | OHIP FEE: "Q005", "Q118", "Q119", "Q120", "Q121", "Q122", "Q123", "Q133" |
| Holter monitoring | CIHI-DAD | CCP: "0354"  CCI: "2HZ24JAKH" |
|  | OHIP | OHIP FEE: "G311", "G320", "G647", "G648", "G649", G650", "G651", "G652", "G653", "G654", "G655", "G656", "G657", "G658", "GG59",  "G660", "G661", "G682", "G683", "G684", "G685", "G686", "G687", "G688", "G689", "G690", "G692", "G693" |
| Parathyroid hormone testing | OHIP | OHIP FEE: “L330” |
| Pulmonary function testing | OHIP | OHIP FEE: "J301", "J303", "J304", "J305", "J306", "J307", "J308", "J309", "J310", "J311", "J313", "J315", "J316", "J317", "J318", "J319", "J320", "J322", "J323", "J324", "J327", "J328", "J330", "J331", "J332", "J333", "J334", "J335", "J340", "J341", "E450", "E451" |
| Urinalysis | OHIP | OHIP FEE: L253, L254, L255, L633 |

**Supplementary Table S5.** Coding definitions for patients receiving maintenance dialysis

| **Characteristic** | **Database** | **Codes** |
| --- | --- | --- |
| Chronic Dialysis (having 2 of these codes separated by at least 90 days, but less than 150 days, to ensure it is truly chronic Dialysis | OHIP | OHIP FEE: "R849", "G323", "G325", "G326", "G860", "G862", "G863", "G865", "G866", "G082", "G083", "G085", "G090", "G091", "G092", "G093", "G094", "G095", "G096", "G294", "G295", "G333", "H540", "H740", "G330", "G331", "G332", "G333", "G861", "G864" |

**Supplementary Table S6.** Operating characteristics of hospital diagnosis codes used to define the primary and secondary outcomes

| **Outcome** | **Outcome component** | **ICD-10/CCI/OHIP/ORGD codes in this study** | **ICD-10 codes used in the validation study** | **Reference Standard** | **Operating Characteristics, % (95% CI)** | | | **Study** |
| --- | --- | --- | --- | --- | --- | --- | --- | --- |
|  |  |  |  |  | **Sensitivity** | **Specificity** | **Positive predictive value** |  |
| **Hospital visit with myelosuppression ^a^** | Aplastic anemia/pancytopenia | ICD-9 codes: 2848, 2849,2859  ICD-10 codes: D611, D612, D613, D619, D618 | ICD-10 codes  D611 | Identification of aplastic anemia in the review of the medical charts of four German hospitals covering the years 2014 and  2015 was done by experienced nurses, pharmacists,  and hospital medical coders from the participating hospitals after having completed a 1-month  training phase. | NA | NA | 74 (60- 85) | **Kuklik**2017^20^ |
|  | Neutropenia | ICD-9 codes: 2880,2889  ICD-10 code: D700, D708, D729 | NA | NA | NA | NA | NA |  |
|  | Thrombocytopenia | ICD-9 codes: 2872,2873,2874,2875,4466  ICD-10 codes: D6938, D696 | ICD-10 codes  D693 | Information from the chart reviews was used to identify patients with chronic idiopathic thrombocytopenic purpura (ITP).  In addition to requiring an ITP diagnosis recorded in the medical charts for a period of more than 6 months, a platelet count below 150 × 10^9^/L was required to confirm the diagnosis | NA | NA | 93 (91-96) | Heden KE 2009^21^ |
| **Hospital visit with pneumotoxicity** |  | ICD-9 codes: 4800, 4801, 4802, 4808, 4809, 481, 4822,4820, 4821, 48240, 48232, 48239, 48282, 48283, 4830, 48289, 4829, 4831, 4838,4848,4847,485,481,514,486, 7700  ICD-10 codes: J12, J13, J14, J15, J16, J17, J18, P23 | ICD-10 codes:  J12, J13, J14, J15, J16, J17, J18, | Chart review was used to identify patients with pneumonia using relevant signs and symptoms consistent with a diagnostic of pneumonia: the documentation of cough,  sputum production, pleuritic chest pain, fever  [37.5 degrees Celsius], shortness of breath, crackles [crepitations],  and aspiration | 98 (97-98) | 97(96-98) | 96 (95-97) | Skull SA 2008^22^ |
| **Hospital visit with hepatotoxicity** |  | ICD-9 codes: 5733, 570, 5728  ICD 10 codes: K711, K716, K719, K720, K729, K759 | ICD-10 codes: K711, K716, K719, K720, K729 | Chart review was used to identify patients with hepatotoxicity and acute liver failure using  predefined criteria. Hepatotoxicity was defined by Alanine aminotransferase >1,000 U/L, and acute liver injury was defined by an international normalized ratio (INR) > 1.5 | 86 (65 -97) | 96 (79 -100) | 95 (75-100) | Myers RP 2007^23^ |
| **Hospital visit with sepsis** |  | ICD-9 codes: 0031,0362,0380,03810,03819,0382,0383,0384,0381,03842,03844,03843,03849,0388,0389  ICD-10 codes: A021, A392, A393, A394, A400, A401, A402, A408, A409, A410, A411, A412, A403, A414, A4159, A413, A4150, A4151, A4152, A4158, A4180, A4188, A427, A419 | ICD-10 codes: A021, A392, A393, A394, A400, A401, A402, A408, A409, A410, A411, A412, A403, A414, A4159, A413, A4150, A4151, A4152, A4158, A4180, A4188, A427, A419 | Chart review was reviewed by two  independent physicians (one trained in intensive care  medicine and the other in surgery) to identify cases of sepsis using a  checklist criteria tool developed based on the  ACCP/SCCM 2001 Consensus Conference updated definitions  and consensus of clinical experts. | 46 (42- 50) | 99 (97-100) | 98 (96-99) | Jolley RJ, 2015^24^ |
| **Death** |  |  |  |  | 98 | 100 |  | Jha P 1996^25^ |

Abbreviations: ICD-10, International Classification of Diseases, Tenth Revision; NA, not available

^a^The algorithm used to identify myelosuppression includes a diagnosis of aplastic anemia/pancytopenia neutropenia or thrombocytopenia. ICD-10 code algorithms that captured neutropenia were not validated. Despite moderate to high positive predictive values for algorithms detecting aplastic anemia and thrombocytopenia, neither sensitivity nor specificity was available.

**Supplementary Table S7.** Baseline characteristics^a^ of adults receiving maintenance dialysis newly prescribed low-dose methotrexate versus those newly prescribed hydroxychloroquine (HCQ) in Ontario, Canada (1997–2020)

|  | | DMARD Cohort (N=462) | | | | Standardized difference^b^ |
| --- | --- | --- | --- | --- | --- | --- |
|  |  | Low -dose methotrexate (n=55) | | Hydroxychloroquine  (n=407) | |  |
| Demographics | | | | | | |
| Age at cohort entry | Mean ± sd | 63.15 | 15.01 | 54.94 | 18.19 | 49% |
|  | Median (iqr) | 66 | (53-73) | 58 | (39-70) | . |
|  | 50-<66 | 27 | 49.1% | 279 | 68.6% | 40% |
|  | 66-<70 | 6 | 10.9% | 26 | 6.4% | 16% |
|  | 70-<75 | 10 | 18.2% | 31 | 7.6% | 32% |
|  | 75-<80 | 6 | 10.9% | 32 | 7.9% | 10% |
|  | 80+ | 6 | 10.9% | 39 | 9.6% | 4% |
| Sex | F | 28 | 50.9% | 291 | 71.5% | 43% |
|  | M | 27 | 49.1% | 116 | 28.5% | 43% |
| Year of cohort entry | 1997-2004 | 8 | 14.5% | 63 | 15.5% | 3% |
|  | 2005-2012 | 19 | 34.5% | 129 | 31.7% | 6% |
|  | 2013-2020 | 28 | 50.9% | 215 | 52.8% | 4% |
| Socio-economic status^c^ | 1 | 9 | 16.4% | 110 | 27.0% | 26% |
|  | 2 | 10 | 18.2% | 105 | 25.8% | 18% |
|  | 3 | 12 | 21.8% | 82 | 20.1% | 4% |
|  | 4 | 15 | 27.3% | 61 | 15.0% | 30% |
|  | 5 | 9 | 16.4% | 49 | 12.0% | 13% |
| Prescriber information |  |  |  |  |  |  |
| Rheumatologist |  | 27 | 49.1% | 182 | 44.7% | 9% |
| Other |  | 16 | 29.1% | 149 | 36.6% | 16% |
| Missing |  | 12 | 21.8% | 76 | 18.7% | 8% |
| Comorbidities^d^ |  |  |  |  |  |  |
| Acute kidney injury |  | 28 | 50.9% | 167 | 41.0% | 20% |
| Angina |  | 20 | 36.4% | 113 | 27.8% | 18% |
| Atrial fibrillation/flutter |  | 10 | 18.2% | 51 | 12.5% | 16% |
| Chronic liver disease |  | 9 | 16.4% | 64 | 15.7% | 2% |
| Hypertension |  | 24 | 43.6% | 112 | 27.5% | 34% |
| Prior myelotoxicity |  | 9 | 16.4% | 84 | 20.6% | 11% |
| Prior liver toxicity |  | 7 | 12.7% | 31 | 7.6% | 17% |
| Prior sepsis |  | 9 | 16.4% | 65 | 16.0% | 1% |
| Anemia |  | 35 | 63.6% | 247 | 60.7% | 6% |
| Coronary artery disease (minus angina) |  | 23 | 41.8% | 186 | 45.7% | 8% |
| Congestive heart failure |  | 27 | 49.1% | 164 | 40.3% | 18% |
| Cirrhosis |  | 7 | 12.7% | 34 | 8.4% | 14% |
| Chronic obstructive pulmonary disease |  | 25 | 45.5% | 189 | 46.4% | 2% |
| Dementia |  | 6 | 10.9% | 28 | 6.9% | 14% |
| Dyslipidemia |  | 16 | 29.1% | 88 | 21.6% | 17% |
| Major hemorrhage |  | 8 | 14.5% | 62 | 15.2% | 2% |
| Hypothyroidysm |  | 7 | 12.7% | 60 | 14.7% | 6% |
| Peripheral vascular disease |  | 6 | 10.9% | 80 | 19.7% | 25% |
| Depression |  | 12 | 21.8% | 73 | 17.9% | 10% |
| Rheumatoid arthritis |  | 27 | 49.1% | 175 | 43.0% | 12% |
| Cancer^e^ |  | 16 | 29.1% | 92 | 22.6% | 15% |
| Prostatic hyperplasia |  | 6 | 10.9% | 20 | 4.9% | 22% |
| Prior pneumotoxicity |  | 13 | 23.6% | 93 | 22.9% | 2% |
| Skin-related infections |  | 37 | 67.3% | 237 | 58.2% | 19% |
| Fractures |  | 6 | 10.9% | 33 | 8.1% | 10% |
| Macula degeneration |  | 6 | 10.9% | 24 | 5.9% | 18% |
| Gastroesophageal reflux disease |  | 24 | 43.6% | 157 | 38.6% | 10% |
| Arrythmia |  | 17 | 30.9% | 72 | 17.7% | 31% |
| Osteoarthritis |  | 6 | 10.9% | 34 | 8.4% | 8% |
| Diabetes |  | 9 | 16.4% | 33 | 8.1% | 26% |
| Urinary tract infection |  | 12 | 21.8% | 104 | 25.6% | 9% |
| Gout |  | 11 | 20.0% | 70 | 17.2% | 7% |
| Other bacterial infections |  | 38 | 69.1% | 244 | 60.0% | 19% |
| Prosthetic joint infection |  | 21 | 38.2% | 142 | 34.9% | 7% |
| Psoriasis |  | 13 | 23.6% | 11 | 2.7% | 65% |
| Dermatomyositis |  | 11 | 20.0% | 202 | 49.6% | 65% |
| Atopic dermatitis or eczema |  | 18 | 32.7% | 140 | 34.4% | 4% |
| Lupus erythematosus |  | 13 | 23.6% | 223 | 54.8% | 67% |
| Charlson comorbidity index^f^ | Mean ± sd | 5.13 | 2.36 | 5.08 | 2.06 | 2% |
|  | Median (iqr) | 5 | (4-6) | 5 | (4-6) | . |
| Medication use^g^, no. (%) |  |  |  |  |  |  |
| Alpha adrenergic blocking agents |  | 6 | 10.9% | 11 | 2.7% | 33% |
| Ace inhibitor |  | 8 | 14.5% | 37 | 9.1% | 17% |
| Anticoagulants |  | 6 | 10.9% | 30 | 7.4% | 12% |
| Angiotensin ii receptor blockers |  | 7 | 12.7% | 26 | 6.4% | 22% |
| Antiplatelet agents |  | 6 | 10.9% | 13 | 3.2% | 30% |
| Beta blockers |  | 20 | 36.4% | 61 | 15.0% | 51% |
| Beta agonist |  | 7 | 12.7% | 11 | 2.7% | 38% |
| Calcium channel blocker |  | 13 | 23.6% | 58 | 14.3% | 24% |
| Glucocorticoids^h^ |  | 19 | 34.5% | 90 | 22.1% | 28% |
| Loop diuretics |  | 13 | 23.6% | 52 | 12.8% | 28% |
| Opioids |  | 7 | 12.7% | 57 | 14.0% | 4% |
| Insulin |  | 7 | 12.7% | 22 | 5.4% | 26% |
| Proton pump inhibitors |  | 15 | 27.3% | 67 | 16.5% | 26% |
| Statins |  | 20 | 36.4% | 75 | 18.4% | 41% |
| Antibiotics |  | 18 | 32.7% | 54 | 13.3% | 47% |
| Number of unique drug names | Mean ± sd | 7.24 | 8.06 | 3.96 | 6.02 | 46% |
|  | Median (iqr) | 2 | (0-16) | 0 | (0-9) | . |
| Number of unique dins | Mean ± sd | 7.95 | 9.02 | 4.33 | 6.65 | 46% |
|  | Median (iqr) | 2 | (0-17) | 0 | (0-9) | . |
| Healthcare use^i^ |  |  |  |  |  |  |
| GP/FP visits | Mean ± sd | 12.89 | 15.15 | 11.08 | 13 | 13% |
|  | Median (iqr) | 9 | (4-16) | 7 | (3-13) | . |
| Nephrology visits | Mean ± sd | 43.95 | 26.29 | 47.97 | 29.88 | 14% |
|  | Median (iqr) | 52 | (26-60) | 52 | (30-56) | . |
| Number of hospitalizations | Mean ± sd | 1.45 | 1.78 | 1.56 | 1.85 | 6% |
|  | Median (iqr) | 1 | (0-2) | 1 | (0-2) | . |
| Number of emergency department visits | Mean ± sd | 2.25 | 2.84 | 2.59 | 3.19 | 11% |
|  | Median (iqr) | 1 | (0-4) | 2 | (0-4) | . |
| TSH test |  | 16 | 29.1% | 118 | 29.0% | 0% |
| Bone mineral density test |  | 7 | 12.7% | 57 | 14.0% | 4% |
| Cardiac stress test |  | 18 | 32.7% | 117 | 28.7% | 9% |
| Carotid ultrasound |  | 7 | 12.7% | 62 | 15.2% | 7% |
| Chest-xray |  | 42 | 76.4% | 327 | 80.3% | 9% |
| Cholesterol test (total cholesterol, hdl) |  | 23 | 41.8% | 136 | 33.4% | 17% |
| Ct abdomen |  | 19 | 34.5% | 109 | 26.8% | 17% |
| Ct extremities |  | 8 | 14.5% | 83 | 20.4% | 16% |
| Ct pelvis |  | 17 | 30.9% | 101 | 24.8% | 14% |
| Ct thorax |  | 10 | 18.2% | 81 | 19.9% | 4% |
| Echocardiography |  | 30 | 54.5% | 221 | 54.3% | 0% |
| Flu shot |  | 21 | 38.2% | 105 | 25.8% | 27% |
| Mammography |  | 6 | 10.9% | 44 | 10.8% | 0% |
| Parathyroid hormone testing |  | 14 | 25.5% | 91 | 22.4% | 7% |
| Pulmonary function test |  | 13 | 23.6% | 82 | 20.1% | 8% |
| Urinalysis |  | 25 | 45.5% | 167 | 41.0% | 9% |
| Hemodialysis |  | 51 | 92.7% | 366 | 89.9% | 10% |

Abbreviations: DMARD,disease-modifying antirheumatic drugs: ; ACE inhibitor, angiotensin-converting-enzyme inhibitor; H2 blockers, Histamine H2-receptor antagonists; eGFR, estimated glomerular filtration rate; IQR, interquartile range; LHIN, Local Health Integration Network; ACR, urine albumin-to-creatinine ratio.

^a^ Unless otherwise specified in the footnotes, baseline characteristics were assessed on the date the patient filled a low-dose methotrexate prescription or a hydroxychloroquine prescription—the cohort entry date.

^b^ The difference between the groups divided by the pooled SD; a value greater than 10% is interpreted as a meaningful difference.^25^

^c^ Income was categorized into fifths of average neighborhood income on the cohort entry date.

^d^ Baseline comorbidities were assessed in the 5-year period before the cohort entry date.

^e^ Cancer includes the following types of cancer: skin, mouth (lip, tonsil, etc), throat, stomach, small/large intestine, liver, gall bladder, pancreas, breast, male/female reproductive organs, heart, lung, bone, urinary system (kidney, bladder, etc), endocrine glands, as well as leukemias and lymphomas

^f^ Presence of kidney disease is a variable in the Charlson comorbidity index, which automatically results in all individuals receiving a minimum score of 2

^g^ Medication use was examined in the 120-day period before the cohort entry date (the Ontario Drug Benefit program dispenses a maximum 100-day supply).

^h^Glucocorticoids included many medications regardless of their route of administration such as hydrocortisone acetate, dexamethasone, beclomethasone dipropionate, prednisone, hydrocortisone, flumetasone pivalate, clioquinol, betamethasone valerate, betamethasone, triamcinolone acetonide, triamcinolone diacetate, triamcinolone, flurandrenolide, betamethasone & dexamethasone sodium phosphate, cortisone acetate, dexamethasone tebutate, prednisolone, dexamethasone, corticotrophin, prednisolone acetate, fluocinolone acetonide, hydrocortisone sodium succinate, methylprednisolone sodium succinate, methylprednisolone acetate, methylprednisolone disodium phosphate, methylprednisolone, fluocinonide, betamethasone disodium phosphate, medrysone & polyvinyl alcohol, prednisolone acetate & sulfacetamide sodium, dexamethasone & neomycin sulfate & polymyxin b sulfate, clioquinol & flumetasone pivalate, clioquinol & hydrocortisone, 1,2-propanediol diacetate & acetic acid & benzethonium chloride & hydrocortisone, clioquinol & triamcinolone acetonide, flurandrenolide, fluocinolone acetonide, dexamethasone & neomycin sulfate, hydrocortisone & lidocaine HCL & neomycin sulfate, hemorrhoidal veinous plexus, prednisone & pheniramine maleate & inositol & phosphatidyl choline & vitamin a & vitamin d2& vitamin e, chloramphenicol & hydrocortisone acetate, haemorroidal veinous plexus, dexamethasone & framycetin sulfate & gramicidin, dibucaine hcl & esculin & framycetin sulfate & hydrocortisone, betamethasone valerate & neomycin sulfate, betamethasone valerate & gentamicin sulfate, prednisolone acetate & sulfacetamide sodium, ascorbic acid & chlorpheniramine maleate & prednisone acetate, neomycin sulfate & prednisolone acetate & sulfacetamide sodium, gramicidin & neomycin sulfate & triamcinolone acetonide, methylprednisolone, acetylsalicylic acid & methyltestosterone, methylprednisolone sulfate & neomycin sulfate, hydrocortisone acetate & neomycin sulfate, aluminum chlorohydrate & methylprednisolone acetate & neomycin sulfate & sulfur, gramicidin & neomycin sulfate & nystatin & triamcinolone acetonide, hydrocortisone acetate & zinc oxide, hydrocortisone acetate & pramoxine hcl & zinc sulfate, aluminum chlorohydrate & methylprednisolone acetate & sulfur, hydrocortisone acetate & zinc oxide, hydrocortisone acetate & pramoxine hcl & zinc sulfate, desonide, clobetasol propionate, beclomethasone dipropionate & clioquinol, bacitracin zinc & hydrocortisone & neomycin sulfate & polymyxin b sulfate, hydrocortisone & neomycin sulfate & polymyxin b sulfate, gramicidin & neomycin sulfate & nystatin & triamcinolone acetonide, fluorometholone & polyvinyl alcohol, aluminum chlorohydrate & methylprednisolone acetate & sulfur, fluorometholone, lidocaine hcl & methylprednisolone acetate, flumetasone pivalate & salicylic acid, fluorometholone, lidocaine hcl & methylprednisolone acetate, aclometasone dipropionate, allantoin & chloramphenicol & hydrocortisone, amcinonide, atropine sulfate & prednisolone acetate, bacitracin & hydrocortisone & neomycin sulfate & polymyxin b sulfate, benzalkonium & dexamethasone & tobramycin , benzocaine & hydrocortisone acetate & zinc sulfate, betamethasone & sulfacetamide sodium, betamethasone acetate & betamethasone sodium phosphate, betamethasone benzoate, betamethasone dipropionate, betamethasone dipropionate &calcipotriene, betamethasone dipropionate &clotrimazole, betamethasone dipropionate & gentamicin sulfate, betamethasone dipropionate & salicylic acid, betamethasone disodium phosphate, betamethasone valerate & salicylic acid, betamethasone valerate & gentamycin sulfate, betamethasone valerate & neomycin sulfate, budesonide, camphor & hydrocortisone & menthol, chlorbutol & dexamethasone &tobramycin, ciclesonide.

^i^Total number of healthcare visits/tests in the 12-month period before the cohort entry date.

**Supplementary Table S8.** Frequency of each component in the cohort

| **Event type** | **Aggregate event rates^a^** | |
| --- | --- | --- |
| Death | 14 | 3.03% |
| Pneumotoxicity or sepsis or myelotoxicity or hepatotoxicity or sepsis | 37 | 8.01% |
| Pneumotoxicity | 18 | 3.90% |
| Sepsis | 17 | 3.68% |
| Myelotoxicity | 18 | 3.90% |
| Hepatotoxicity | 0 | 0% |

^a^ These categories are not mutually exclusive

**Supplementary Table 9. Risk of an outpatient or hospital visit with a thyroid disorder in adults receiving maintenance dialysis within 90 days of starting a new prescription for low-dose methotrexate vs hydroxychloroquine ^a^**

|  | **Low-dose**  **methotrexate**  **(n = 55)** | **Hydroxy-chloroquine**  **(n = 407)** | **Unadjusted RD**  **(95% CI)** | **Unadjusted**  **RR**  **(95% CI)** | **Adjusted**  **RD, %**  **(95% CI)^b^** | **Adjusted NNH**  **(95% CI)** | **Adjusted**  **RR (95% CI)^b^** |
| --- | --- | --- | --- | --- | --- | --- | --- |
| A receipt of a thyroid test during an outpatient or hospital visit^c^ |  |  | -1.82 (-9.23 to 5.59) | 0.80 (0.30 to 2.16) | -2.61 (-7.54 to 2.32) | NA | 0.88 (0.35 to 2.24) |

Abbreviations: NA, not applicable; NNH, number needed to harm.

^a^ Reference group: Hydroxychloroquine.

^b^ Due to the small sample of methotrexate users, the regression analyses were adjusted for only 3 key risk factors—age (years), sex (male/female), and the number of unique drugs prescribed in the 120 days before cohort entry—to reduce the risk of overfitting. This approach aligns with the traditional recommendation of limiting the number of covariates relative to the number of outcome events (e.g., 10 events per variable).

^C^To comply with ICES privacy regulations to minimize the risk of identification, specific values of cells with ≤ 5 adults were suppressed (reported as <6). As a result, the number of individuals who received a thyroid test during an outpatient or hospital visit is not presented in the table.

**Supplementary Table 10.** Risk of death or a hospital visit with myelosuppression, sepsis, pneumotoxicity, or hepatotoxicity in adults receiving maintenance dialysis within 90 days of starting a new prescription for low-dose methotrexate vs hydroxychloroquine after adjustement for 55 baseline characteristics ^a^

|  | Events, n (%) |  |  |
| --- | --- | --- | --- |
|  | **Low-dose**  **methotrexate**  **(n = 55)** | **Hydroxy-chloroquine**  **(n = 407)** | **Adjusted**  **RR (95% CI)^b^** |
| **Primary outcome** |  |  |  |
| Death or hospital visit with myelosuppression, sepsis, pneumotoxicity, or hepatotoxicity^c^ | 16 (29.1) | 29 (7.1) | 4.15  (1.60 to 10.76) |

^a^ Reference group: Hydroxychloroquine.

^b^ As a sensitivity analysis, the regression models were adjusted for 55 baseline variables with standardized differences >10% (i.e., variables that were not balanced between the low-dose methotrexate and hydroxychloroquine groups): Age at cohort entry, Sex, Socio-economic status, Prescriber information, Acute kidney injury, Angina, Atrial fibrillation/flutter, Hypertension, Prior myelotoxicity, Prior liver toxicity, Congestive heart failure, Cirrhosis, Dementia, Dyslipidemia, Peripheral vascular disease, Depression, Rheumatoid arthritis, Cancer, Prostatic hyperplasia, Skin-related infections, Fractures, Macular degeneration, Gastroesophageal reflux disease, Arrhythmia, Diabetes, Other bacterial infections, Lupus erythematosus, Dermatomyositis, Alpha adrenergic blocking agents, ACE inhibitors, Anticoagulants, Angiotensin II receptor blockers, Antiplatelet agents, Beta blockers, Beta agonists, Calcium channel blockers, Glucocorticoids, Insulin, Proton pump inhibitors, Statins, Antibiotics, Number of unique drug names, Number of unique DINs, GP/FP visits, Nephrology visits, Number of hospitalizations, Number of emergency department visits, Flu shot, Cholesterol test, CT abdomen, CT extremities, CT pelvis. This sensitivity analysis aimed to assess the robustness of the primary findings. While this approach improves adjustment for baseline imbalances, we acknowledge that including a large number of variables relative to the number of events may introduce model instability and overfitting

**Supplementary Figure S1.** Study design diagram comparing the use of low-dose methotrexate versus hydroxychloroquine.

The diagram summarizes the cohort creation steps, including inclusion criteria, exposure definition, washout period, follow-up, censoring, and outcome assessment

Abbreviations: DMARD, disease-modifying antirheumatic drug; LD-MTX, low-dose methotrexate; HCQ, hydroxychloroquine

**Supplementary Figure S2**. E-value Analysis to Assess the Extent of Unmeasured Confounding That Would Be Required to Negate the Observed Results

E-value for point estimate: 5.73 and for lower bound of the confidence interval: 2.9

**Supplementary References**

1.	Shively RM, Nogar J. A need for consensus guideline update: recommend explicit restriction of methotrexate in end-stage renal patients. *Rheumatology*. 2020;59(11):e93-e94.

2.	Basile C, Montanaro A, Semeraro A. Should low‐dose methotrexate therapy be prescribed to dialysis patients? *Nephrology Dialysis Transplantation*. 2002;17(3):530-531.

3.	Boulanger H, Launay-Vacher V, Hierniaux P, Fau J-B, Deray G. Severe methotrexate intoxication in a haemodialysis patient treated for rheumatoid arthritis. *Nephrology Dialysis Transplantation*. 2001;16(5):1087-1087.

4.	Chatham WW, Morgan SL, Alarcón GS. Renal failure: a risk factor for methotrexate toxicity. *Arthritis & Rheumatism: Official Journal of the American College of Rheumatology*. 2000;43(5):1185-1186.

5.	Seneschal J, Héliot-Hostein I, Taieb A. Pancytopenia induced by low-dose methotrexate in a haemodialysis patient treated for bullous pemphigoid. *Journal of the European Academy of Dermatology and Venereology: JEADV*. 2007;21(8):1135-1136.

6.	Aristizabal-Alzate A, Nieto-Rios JF, Ocampo-Kohn C, Serna-Higuita LM, Bello-Marquez DC, Zuluaga-Valencia GA. Successful multiple-exchange peritoneal Dialysis in a patient with severe hematological toxicity by methotrexate: case report and literature review. *Brazilian Journal of Nephrology*. 2018;41:427-432.

7.	Chess JA, Scholey G, Mikhail AI. Neutropenia associated with the use of low-dose methotrexate in a peritoneal dialysis patient. *Nephrology Dialysis Transplantation*. 2004;19(8):2159-2159.

8.	Cheung KKT, Chow KM, Szeto CC, Tai MHL, Kwan BCH, Li PKT. Fatal pancytopenia in a hemodialysis patient after treatment with low-dose methotrexate. *JCR: Journal of Clinical Rheumatology*. 2009;15(4):177-180.

9.	Ellman MH, Hou S, Ginsberg D. Low‐dose methotrexate and severe neutropenia in patients undergoing renal Dialysis. *Arthritis & Rheumatism: Official Journal of the American College of Rheumatology*. 1990;33(7):1060-1061.

10.	Liu H, Liu F, Zhang M, Yan W, Sang H. Combined acute interstitial pneumonitis and pancytopenia induced by low-dose methotrexate in a hemodialysis patient treated for bullous pemphigoid. *Anais brasileiros de dermatologia*. 2015;90:43-45.

11.	Liu WC, Chen HC, Chen JS. Clinical Dilemma Over Low-dose Methotrexate Therapy in Dialysis Patients A Case report and Review of Literature. 2014;

12.	Mima A, Nagahara D, Tansho K. Methotrexate induced pneumatosis intestinalis under hemodialysis patient. *Hemodialysis International*. 2017;21(1):E9-E12.

13.	Sun C, Lin H, Chen Y, Tsai C, Wu M. Leukemoid reaction after methotrexate-induced pancytopenia in a patient undergoing continuous ambulatory peritoneal Dialysis. *Chang Gung medical journal*. 2006;29(5):513.

14.	Boey O, Van Hooland S, Woestenburg A, Van der Niepen P, Verbeelen D. Methotrexate should not be used for patients with end-stage kidney disease. *Acta Clinica Belgica*. 2006;61(4):166-169.

15.	Yang C-P, Kuo M-C, Guh J-Y, Chen H-C. Pancytopenia after low dose methotrexate therapy in a hemodialysis patient: case report and review of literature. *Renal failure*. 2006;28(1):95-97.

16.	Diskin CJ, Stokes TJ, Dansby LM, Radcliff L, Carter TB. Removal of methotrexate by peritoneal Dialysis and hemodialysis in a single patient with end-stage renal disease. *The American journal of the medical sciences*. 2006;332(3):156-158.

17.	Abdullah S, Al-Mashgari S, Elnour S, Elhag S. Myelosuppression Following Low Dose Methotrexate in Peritoneal Dialysis Patient - a Case Report. Conference Abstract. *European Journal of Case Reports in Internal Medicine*. 2023;10(Supplement 1):522-523.

18.	Ouellette S, Shah R, Razi S, Ashforth G, Wassef C. Fatal low‐dose methotrexate toxicity: A case report and literature review. *Dermatologic Therapy*. 2022;35(12):e15945.

19.	Coroner’s Inquest into the Death Of A Hemodialysis Patient On Methotrexate. Accessed November 1, 2024. <https://pharmacyconnection.ca/coroners-inquest-fall-2018/>

20. Kuklik N, Stausberg J, Jöckel KH. Adverse drug events in German hospital routine data: A validation of International Classification of Diseases, 10th revision (ICD-10) diagnostic codes. PLoS One. 2017 Nov 2;12(11):e0187510. doi: 10.1371/journal.pone.0187510. PMID: 29095926; PMCID: PMC5667751.

21. Heden KE, Jensen AØ, Farkas DK, Nørgaard M. Validity of a procedure to identify patients with chronic idiopathic thrombocytopenic purpura in the Danish National Registry of Patients. Clin Epidemiol. 2009 Aug 9;1:7-10. doi: 10.2147/clep.s4832. PMID: 20865080; PMCID: PMC2943173.

22. Skull SA, Andrews RM, Byrnes GB, Campbell DA, Nolan TM, Brown GV, Kelly HA. ICD-10 codes are a valid tool for identification of pneumonia in hospitalized patients aged > or = 65 years. Epidemiol Infect. 2008 Feb;136(2):232-40. doi: 10.1017/S0950268807008564. Epub 2007 Apr 20. PMID: 17445319; PMCID: PMC2870806.

23. Myers RP, Leung Y, Shaheen AA, Li B. Validation of ICD-9-CM/ICD-10 coding algorithms for the identification of patients with acetaminophen overdose and hepatotoxicity using administrative data. BMC Health Serv Res. 2007 Oct 2;7:159. doi: 10.1186/1472-6963-7-159. PMID: 17910762; PMCID: PMC2174469.

24. Jolley RJ, Quan H, Jetté N, Sawka KJ, Diep L, Goliath J, Roberts DJ, Yipp BG, Doig CJ. Validation and optimisation of an ICD-10-coded case definition for sepsis using administrative health data. BMJ Open. 2015 Dec 23;5(12):e009487. doi: 10.1136/bmjopen-2015-009487. PMID: 26700284; PMCID: PMC4691777.

25. Jha P, Deboer D, Sykora K, Naylor CD: Characteristics and mortality outcomes of thrombolysis trial participants and nonparticipants: A population-based comparison. J Am Coll Cardiol 27: 1335– 1342, 1996.
